# Identification of Basic Fibroblast Growth Factor as the Dominant Protector of Laminar Shear Medium from the Modified Shear Device in Tumor Necrosis Factor-α Induced Endothelial Dysfunction

**DOI:** 10.3389/fphys.2017.01095

**Published:** 2018-01-05

**Authors:** Huang-Joe Wang, Wan-Yu Lo

**Affiliations:** ^1^Department of Internal Medicine, School of Medicine, China Medical University, Taichung, Taiwan; ^2^Cardiovascular Research Laboratory, Division of Cardiovascular Medicine, Department of Internal Medicine, China Medical University and Hospital, Taichung, Taiwan; ^3^Cardiovascular and Translational Medicine Laboratory, Department of Biotechnology, Hungkuang University, Taichung, Taiwan; ^4^Bachelor Degree Program in Animal Healthcare, Hungkuang University, Taichung, Taiwan

**Keywords:** laminar shear stress, basic fibroblast growth factor, endothelial dysfunction, tumor necrosis factor-α, cytokine antibody array

## Abstract

**Background and Aims:** Endothelial dysfunction is a hallmark of cardiovascular diseases. The straight region of an artery is protected from atherosclerosis via its laminar blood flow and high shear stress. This study investigated the cytoprotective effects of a new laminar shear medium (LSM) derived from a modified cone-and-plate shear device and identified basic fibroblast growth factor (bFGF) secreted by human aortic endothelial cells (HAECs) as the dominant protective factor in the LSM.

**Methods:** Based on a modified cone-and-plate shear device system, HAECs were exposed to laminar shear (15 dynes/cm^2^) and static control for 24 h to produce a new supernatant LSM and static medium (SM). Evaluation of the protective effects of LSM and SM on endothelial dysfunction induced by tumor necrosis factor (TNF)-α (10 ng/mL), which leads to production of reactive oxygen species (ROS), inflammatory monocyte adhesion, and tissue factor activity. ROS induction-, inflammation-, and thrombosis-related genes and protein expression were evaluated by quantitative-PCR and western blotting. To identify the cytokines that played a key role in the cytoprotective action of the LSM, we used cytokine antibody arrays, selected an abundant marker cytokine, bFGF, and validated the different cytoprotective effects of recombinant bFGF (rbFGF) and neutralization by monoclonal antibody (rbFGF+Ab) co-treatment. Aortic and lung tissues from different groups of C57BL/6J mice were examined by immunohistochemistry. SB203580 (specific inhibitor of p38) and BIX02189 (specific inhibitor of MEK5) were used to identify bFGF as the main cytoprotective factor acting via p38/MAPK and MEK5-KLF2 pathways.

**Results:** Compared with traditional LSM, the new LSM not only significantly decreased TNF-α-induced intracellular adhesion molecule 1 and plasminogen activator inhibitor type 1 gene expression, but also significantly increased heme oxygenase 1 gene expression. The new LSM and bFGF attenuated TNF-α-induced ROS induction, inflammation, and tissue factor activity and inhibited the inflammatory- and thrombosis-related gene/protein overexpression both *in vitro* and *in vivo*. Mechanistically, the cytoprotective action of bFGF was mediated via the p38/MAPK and MEK5-KLF2 pathways.

**Conclusion:** bFGF was identified as the critical factor mediating the cytoprotective effects of LSM derived from the modified laminar shear system.

## Introduction

Endothelial cells are constantly exposed to blood flow; shear stress for blood flow within a vessel is defined as τ = 32•μ•Q/π•d^3^, where Q is the mean volumetric flow rate, μ is the mean velocity, and d is the vessel diameter (Papaioannou and Stefanadis, 2005). Previous studies revealed increased oxidative stress, impaired vasodilatation, proinflammatory, and prothrombotic effects on endothelial cells under disturbed low flow stress stresses (<4 dynes/cm^2^). In contrast, laminar flow with high shear stresses (10–70 dynes/cm^2^) are atheroprotective, and they are associated with the following phenotypes: low expression of adhesion molecules/inflammatory genes/chemokine genes, high expression of antioxidant genes, and inhibition of leukocyte adhesion/platelet aggregation/thrombosis (Chiu and Chien, 2011). Most *in vitro* and *in vivo* studies have focused on the effects of different shear stresses (static, laminar flow, and disturbed flow) on underlying endothelial cells (Chien, 2007; Chiu and Chien, 2011; Tarbell et al., 2014).

In *in vitro* shear stress systems, parallel-plate flow chambers and cone-and-plate shear devices are commonly used shear device systems (Rezvan et al., 2011). The original cone-and-plate system has been modified by numerous groups, including Dr. Hanjoong Jo, whose modification of the design included a 10-cm tissue culture disc on which human umbilical vein endothelial cells (HUVECs) were exposed to laminar or oscillating shear stress from a rotating cone, and the angular separation between the cone surface and culture disc was 0.5°. Using this traditional shear device (15 dynes/cm^2^ laminar shear stress, LSS), we observed that human aortic endothelial cells (HAECs) in the center of the culture disc, compared with those at the periphery, were easily detached on the culture disc and did not show the typical high shear stress-induced alignment of endothelial cell shape, likely because of the non-uniform shear stress levels of this device (Rezvan et al., 2011). Therefore, we developed a modified cone-and-plate system to exclude centrally growing HAECs, in which the seeded endothelial cells did not fully cover the 10-cm culture dish, and retained a central circle devoid of endothelial cell growth to avoid interference from dead cells and metabolites contaminating the laminar shear medium (LSM).

For the past several decades many research groups have used various screening approaches to obtain the global genomic profile of the underlying endothelial cells under shear stress using microarray (Chen et al., 2001; McCormick et al., 2001; Dekker et al., 2002). These experiments have been invaluable for identifying novel target genes in cardiovascular studies. However, such approaches failed to reveal important post-transcriptional protein control mechanisms in the endothelium. In the past decade, some studies have conducted proteome analysis of cultured vascular endothelial cells from bovine, rat, mice, and humans under different shear stresses (Wang et al., 2007; Freed and Greene, 2010; Firasat et al., 2014). However, the biological role of the secretome in LSM from underlying endothelial cells exposed to the LSS remains unclear.

Cytokines have been widely studied in the field of biomedicine. In addition to playing critical roles in many normal cellular events, cytokines are involved in the initiation and development of nearly every major life-threatening disease. Cytokines have been explored as potential disease and physiological biomarkers, and cytokine antibody arrays are effective tools for biomarker discovery with high-throughput detection of many proteins simultaneously (Huang, 2007; Wilson, 2015).

Many previous studies reported that tumor necrosis factor-α (TNF-α)-induction as the major indicator of atherogenesis and inflammation in endothelial cells (Blake and Ridker, 2002; Branen et al., 2004). In this study, we demonstrated that LSM derived from a new LSS system had unexpected protective effects in TNF-α-induced endothelial dysfunction [reactive oxygen species (ROS) induction, inflammation, and thrombosis]. Thus, we hypothesized that underlying HAECs secreting cytokines were dominant protectors of LSM, and they possess significant cytoprotective effects. The high abundance HAEC-secreted marker cytokines were selected using Human Cytokine Antibody Array, and autocrine basic fibroblast growth factor (bFGF) was identified as a critical protector in the LSM.

## Materials and methods

### Cell culture, shear apparatus, and LSM/SM collection

HAECs were purchased from Cell Applications, Inc. (San Diego, CA, USA) and cultured in endothelial cell growth medium (Cell Applications, Inc.) according to the manufacturer's recommendations. The human monocytic cell line THP-1 was obtained from the American Type Culture Collection (Manassas, VA, USA) and maintained as previously described (Wang et al., 2014). Using a cone-and-plate shear device, an LSM was obtained when LSS (15 dynes/cm^2^) was applied to HAECs (between passages 2 and 5) for 24 h. A HAEC monolayer fully covering a 10-cm tissue culture dish represents the traditional shear system, whereas a monolayer grown in a 10-cm tissue culture dish excluding a central circle (diameter of 5.41 cm) represents our new shear system. In this study, both systems were exposed to an arterial level of unidirectional laminar shear for 24 h by rotating a Teflon cone using the magnetic stirrer (Supplementary Data 1: video of laminar shear, 15 dynes/cm^2^) and then traditional and new LSM were collected. The static medium (SM) was collected from the same cells exposed to static conditions for 24 h. The collected new LSM and SM were filtered through 0.45-μm filters and stored at −80°C until analysis.

### MTS cell viability assay

Cell viability was measured using the 3-(4,5-dimethylthiazol-2-yl)-5-(3-carboxymethoxyphenyl)-2-(4-sulfo-phenyl)-2H-tetrazolium (MTS) assay, CellTiter 96® AQueous One Solution reagent (Promega, Madison, WI, USA), and absorbance measurement at 490 nm. The viabilities of the control cell groups incubated with fresh medium for 24 h were set to 100%. Cell viabilities of different percentages of LSM-treated cells were compared with those of the individual control group.

### *In Vitro* experiments

For *in vitro* studies, co-treatment of the cells with recombinant human TNF-α (TNF-α) (10 ng/mL, Sigma–Aldrich, St. Louis, MO, USA) and 20% LSM (TNF-α+LSM group), TNF-α and 20% SM (TNF-α+SM group), and TNF-α and recombinant human bFGF (10 ng/mL, Thermo Fisher Scientific, Waltham, MA, USA) (TNF-α+rbFGF group) were performed. Additionally, for the TNF-α+LSM+Ab group, the LSM was pre-neutralized with anti-human bFGF antibody (10 μg/mL, Santa Cruz Biotechnology, Dallas, TX, USA) at 37°C for 1 h before co-treatment with TNF-α. LSM pre-neutralized with anti-IgG secondary antibody (10 μg/mL, Jackson ImmunoResearch, West Grove, PA, USA) at 37°C for 1 h before co-treatment with TNF-α, and this group was named as the TNF-α+LSM+IgG group. After 6 h incubation, genetic analysis, protein expression level measurement, and multiple biochemical experiments were carried out to explore the cytoprotective efficacies of the new LSM.

### Real-time quantitative polymerase chain reaction (qPCR)

All mRNA transcript levels of HAECs were analyzed by qPCR. The individual Universal ProbeLibrary probe and primer sequences for glyceraldehyde-3-phosphate dehydrogenase (*GAPDH*), Kruppel-like factors (*KLF2*), NAD(P)H quinone dehydrogenase 1 (*NQO-1*), heme oxygenase-1 (*HO-1*), thrombomodulin (TM), Tissue factor (*TF*), plasminogen activator inhibitor type 1(*PAI-1*), Kelch-like ECH-associated protein 1 (*Keap-1*), vascular cell adhesion protein 1 (*VCAM-1*), intercellular adhesion molecule 1 (*ICAM-1*), monocyte chemo-attractant protein-1 (*MCP-1*), hepatocyte growth factor (*HGF*), granulocyte-colony stimulating factor (*G-CSF*), IL-17, granulocyte-macrophage colony-stimulating factor (*GM-CSF*), epidermal growth factor receptor (*EGFR*), monokine induced by gamma interferon (*MIG*), and basic fibroblast growth factor (*bFGF*) genes are provided in Supplementary Data 2. All PCRs were performed using the StepOnePlus Real-Time PCR instrument (Applied Biosystems, Foster City, CA, USA). Real time qPCR conditions were defined according to the manufacturer's recommendations. Gene expression levels were analyzed using StepOne software v2.2.

### Western blot analysis

Protein expression levels in the HAECs were analyzed by western blotting as previously described (Wang et al., 2010). Primary antibodies against HO-1 (1:1,000) (Abcam, Cambridge, UK), ICAM-1 (1:500) (Cell Signaling Technology, Danvers, MA, USA), PAI-1 (1:1,000) (Santa Cruz Biotechnology), bFGF (1:500) (Millipore, Billerica, MA, USA), and GAPDH (1:5,000) (Santa Cruz Biotechnology) were used. Immunostaining was visualized using SuperSignal West Pico Chemiluminescent Substrate for HO-1 and PAI-1 and SuperSignal West Femto Maximum Sensitivity Substrate for ICAM-1 and bFGF (Thermo Scientific).

### Measurement of ROS induction

ROS accumulation was detected using 2′,7′-dichlorofluorescin diacetate (H_2_DCFDA, Sigma). After different treatments, all groups were incubated with H_2_DCFDA (10 μM) at 37°C for 30 min in the dark. Unbound H_2_DCFDA was removed by washing with 1× phosphate-buffered saline, and H_2_DCFDA fluorescence was imaged using a fluorescent microscope equipped with a digital camera (Olympus DP72, Tokyo, Japan). The fluorescence intensity (ROS activity) was measured using ImageJ software (ImageJ, National Institute of Health, Bethesda, MD, USA) and expressed as fold-changes of the corresponding control.

### TF activity assay

Cellular TF-mediated procoagulant activity was measured using commercial Tissue Factor Human Chromogenic Activity Assay Kit (Cot. No. ab108906, Abcam) according to the manufacturer's instructions. Serum-starved HAECs (1 × 10^5^ cells) were grown in six-well plates. After different treatments, cells were washed twice with 1× phosphate-buffered saline, followed by incubation with human factor VIIa (FVIIa) and factor X (FX) at 37°C, which allowed the formation of a TF/FVIIa complex at the cell surface. The TF/FVIIa complex converted human FX to factor Xa, which was measured by its ability to metabolize a chromogenic substrate. A standard curve with lapidated human TF was obtained to ensure that measurements were acquired in the linear range of detection.

### Monocyte adhesion assay

In adhesion experiments, THP-1 cells were labeled with calcein acetoxymethyl ester (Calcein-AM; Molecular Probes, Eugene, OR, USA) as previously described (Lo et al., 2017).

### Cytokine antibody array

Semi-quantitative detection of 120 human cytokine and chemokine levels in the SM and LSM was performed using RayBio C-series Human Cytokine Antibody Array C1000 (AAH-CYT-1000-4, RayBiotech, Norcross, GA, USA) according to the manufacturer's instructions as previously described (Zhou et al., 2005). Detection of all spots was performed with the ChemiDoc MP Imaging Systems (Bio-Rad, Hercules, CA, USA), and the intensity of dots was quantified by densitometric analysis and ImageJ software. For each spot, the raw numerical densitometry data were extracted and subjected to background subtraction before normalizing the signal for each cytokine to the positive control spots.

### Animal experimental protocols

Male C57BL/6J mice (8 weeks of age) were used. All animal protocols were approved by the Institutional Animal Care and Use Committee of Hungkuang University (HK-105-29). Mice were randomized into four groups: TNF-α, TNF-α+LSM, TNF-α+LSM+Ab, and TNF-α+rbFGF groups. Intraperitoneal injection of recombinant mouse TNF-α (2 μg/100 μL saline/mouse) and 100 μL of LSM, LSM+Ab, or rbFGF (3 μg/mouse) were administered. After 24 h, mice were sacrificed by CO_2_ narcosis, and the aortic and lung tissues were removed. Paraffin sections (5 μm thickness) were prepared for immunohistochemistry staining.

### Immunohistochemical staining

All aortic tissue cross-sections from the above experiments were prepared with a Bond-Max autostainer (Leica Microsystems, Wetzlar, Germany). Slides were stained with primary antibody on a fully automated Bond-Max system and VBS Refine polymer detection system (Leica Microsystems) as previously described (Lo et al., 2017). Negative controls did not contain primary antibody. Positive immunoreactivity signals in the endothelial layers were measured by ImageJ software. Immunoreactivity signals in endothelial layers from TNF-α and the TNF-α+LSM, TNF-α+LSM+Ab, or TNF-α+rbFGF groups were quantified as previously described (Federici et al., 2002).

### Identification of protective pathways

The cells were pretreated with 10 μM SB203580 (specific inhibitor of p38) and 10 μM BIX02189 (specific inhibitor of MEK5) (Cayman Chemical, Ann Arbor, MI, USA) for 1 h, followed by addition of TNF-α (10 ng/mL) and rbFGF (10 ng/mL) for 6 h. Gene expression was determined by q-PCR as described above.

### Statistical analyses

Independent experiments were conducted to assess the significance of differences between the control group and other groups or TNF-α group and other groups. Significant differences were determined using Student's *t*-test and defined as *p* < 0.05.

## Results

### Development of new LSS system and evaluation of protective effects of LSM

**Figure 1:**
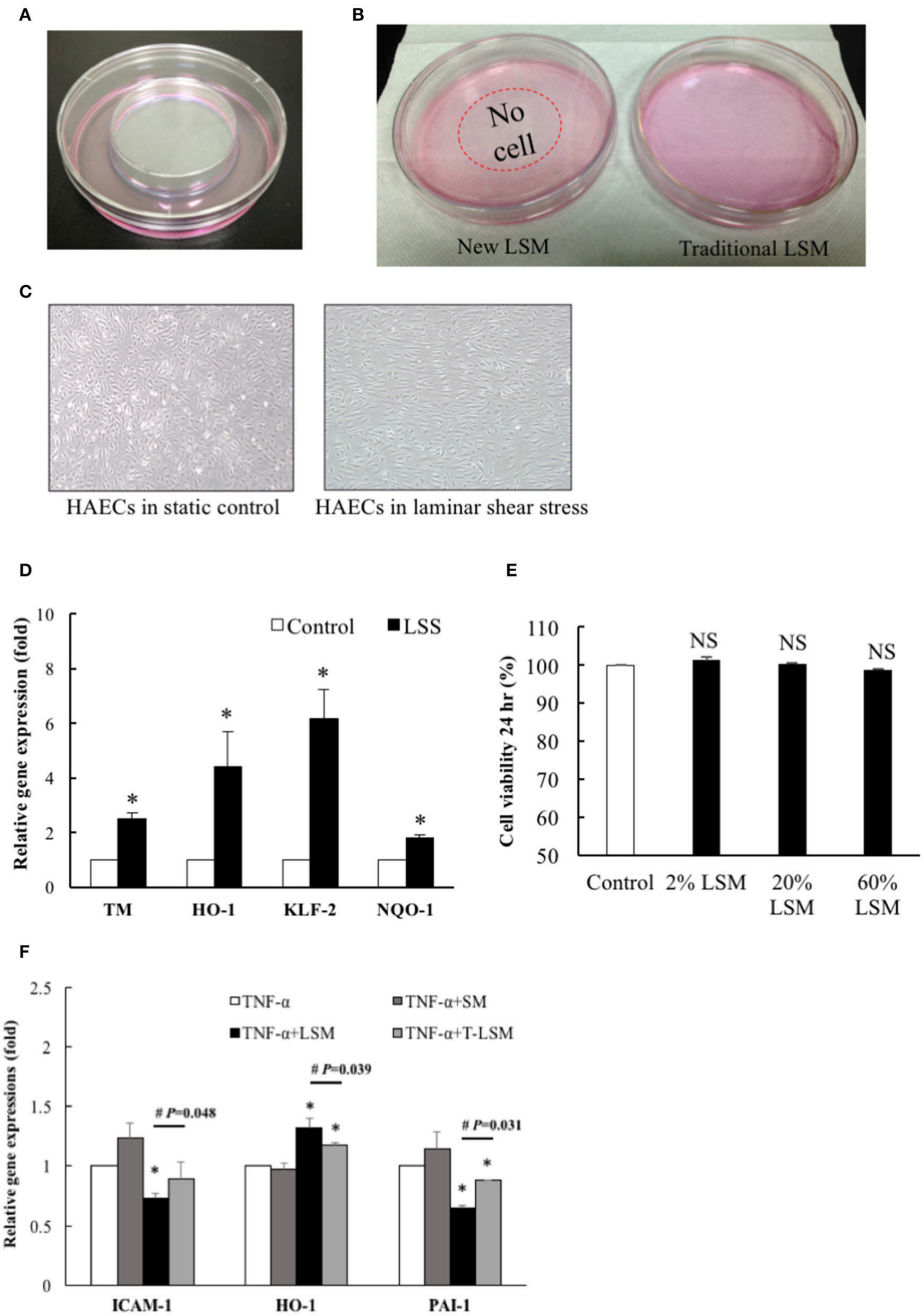
HAECs exposed to LSS from a modified cone-and-plate shear. **(A)** The new LSS system was designed with a central empty circle (diameter is 5.14 cm) in a 10-cm dish, surrounded by cultured HAECs. **(B)** After exposure to shear stress (15 dynes/cm^2^) from the modified cone-and-plate shear device for 24 h, the new LSM showed different phenotypes: medium of the traditional LSS was less clear (right), while the medium of the new LSS system was clearer (left). **(C)** Morphology of HAECs exposed to the new LSS or static conditions for 24 h induced alignment of the cell shape in the direction of the laminar flow (right), while static cultured cells showed a typical polygonal “cobblestone shape” (left). **(D)** HAECs were exposed to the new LSS or static control for 24 h, and the expression levels of *TM, HO-1, NQO-1*, and *KLF-2* in the new LSS-induced cells were increased compared to those in the static control group. *n* = 5. ^*^p < 0.05. **(E)** New LSM, 2, 20, or 60%, was mixed with fresh HAEC medium to test its cytotoxicity. An MTS assay was performed after 24 h incubation. *n* = 3. NS, not significant. **(F)** The new LSM not only significantly inhibited TNF-α (10 ng/mL, 6 h)-induced *ICAM-1* and *PAI-1* gene expression, but also significantly increased the expression of the antioxidant *HO-1* gene compared to traditional LSM. Data are expressed as the mean ± S.E.M. (*n* = 5). ^*^p < 0.05 indicates a significant difference relative to the individual control group. #*p* < 0.05 indicates a significant difference relative to the individual new LSM group. SM, static medium; LSM, new laminar shear medium; T-LSM, traditional laminar shear medium.

We developed a new LSS system. HAECs (1 × 10^6^ cells/dish) were seeded overnight into a 10-cm dish keeping a central empty area (diameter of 51.4 mm) using a 6-cm dish and 2% agarose (Figure 1A). After applying shear stress (15 dynes/cm^2^) for 24 h, the media showed different phenotypes: LSM of traditional LSS was less clear (right), while the LSM of the new LSS system was relatively clear (left) (Figure 1B). HAECs exposed to LSS or static conditions for 24 h showed alignment of the cell shape in the direction of the laminar flow (right), while static cells showed the typical polygonal “cobblestone shape” (left) (Figure 1C). Previous studies suggested that laminar shear increases the expression of mechanosensitive genes to maintain endothelial hemostasis (Chiu and Chien, 2011). In our new LSS system, after HAECs were seeded and exposed to LSS for 24 h, the expression levels of mechanosensitive genes, *TM, HO-1, NQO-1*, and *KLF-2*, in underlying HAECS were upregulated significantly (Figure 1D). Other HAECs were also incubated in fresh media mixed with 2, 20, and 60% LSM for 24 h, but there was no significant induction of cell death (Figure 1E).

The cytokine TNF-α is an important mediator of acute inflammatory processes that occur during the progression of atherosclerosis. Examples include transcriptional regulation of various inflammation- and thrombosis-related genes (Matsumoto et al., 1998; Chiu et al., 2004). Numerous antioxidant pathways are involved in cellular redox homeostasis, among which the nuclear factor-E2-related factor 2 (Nrf2)/Kelch-like ECH-associated protein 1/antioxidant response element signaling pathway is perhaps the most prominent. Oxidative stress causes Nrf2 to dissociate from Kelch-like ECH-associated protein 1, and it translocates into the nucleus to bind to the antioxidant response element and regulate the transcription of downstream target antioxidant genes, such as HO-1 and NQO-1 (Chen et al., 2015). Thus, we treated the other strain of HAECs with TNF-α (10 ng/mL) for 6 h as a positive control platform of endothelial dysfunction. The new LSM not only decreased TNF-α-induced inflammation and thrombosis-related ICAM-1 and PAI-1 gene expression, but also significantly increased antioxidant HO-1 gene expression compared to the traditional LSM (Figure 1F). Thus, the following experiments used the new LSM collected from the modified cone-and-plate shear device.

### Evaluation of protective efficacy of LSM and SM on TNF-α-induced endothelial dysfunction

**Figure 2:**
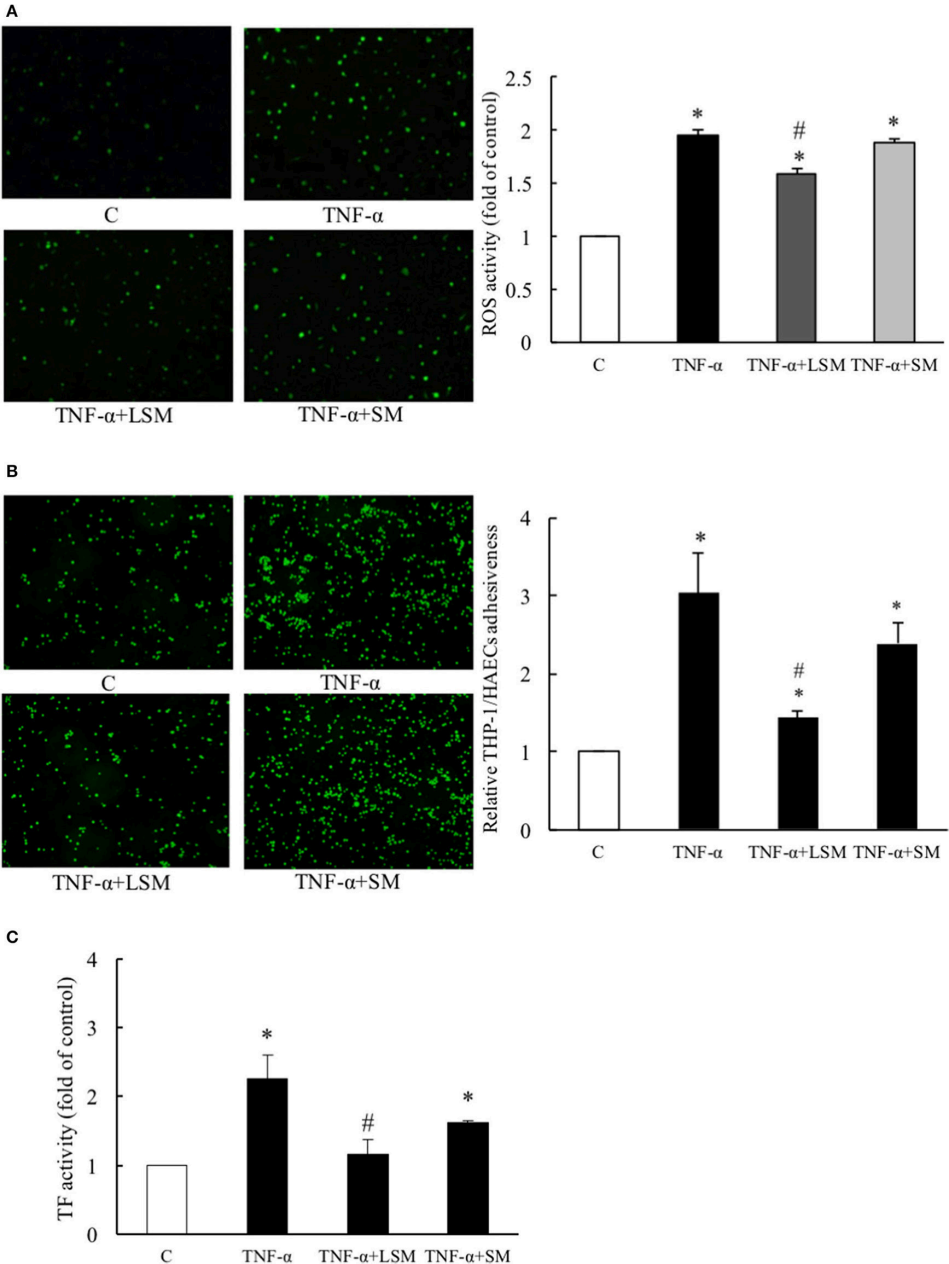
Evaluation of protective efficacies of LSM and SM on TNF-α-induced ROS activity, inflammation, and thrombosis. **(A)** TNF-α group showed significantly intensified H_2_DCFDA dependent fluorescence (average 1.95-fold) compared to in the control group. The TNF-α+LSM group showed significantly attenuated expression (average 1.59-fold) compared to in the control group. **(B)** TNF-α group showed significantly higher (average 3.04-fold) THP-1/HAECs adhesiveness than the control group. The TNF-α+LSM group was significantly attenuated (average 1.44-fold) compared to the control group. **(A,B)** Data are expressed as the mean ± S.E.M. (*n* = 4). ^*^p < 0.05 indicates a significant difference relative to the control group. #*p* < 0.05 indicates a significant downregulation relative to the individual TNF-α group. **(C)** TF activity was significantly induced (average 2.26-fold) in the TNF-α group and attenuated significantly (average 1.16-fold) in the TNF-α+LSM group compared to that in the TNF-α group. Data are expressed as the mean ± S.E.M. (*n* = 3). ^*^p < 0.05 indicates a significant difference relative to the control group. #*p* < 0.05 indicates a significant downregulation relative to the individual TNF-α group. SM, static medium. LSM, new laminar shear medium.

A previous study showed that TNF-α induces a pro-oxidant environment in a cell that can be measured by H_2_DCFDA detection in the intracellular oxidative milieu (Shanmugam et al., 2016). Thus, we evaluated the protective efficacies of LSM and SM using the H_2_DCFDA assay. As shown in Figure 2A, untreated control cells displayed very low levels of H_2_DCFDA-dependent fluorescence. However, the TNF-α group showed a significantly higher average fluorescence intensity (1.95-fold) compared to the control group. The TNF-α+LSM group showed significant attenuation with an average intensity of 1.59-fold relative to the control group. The TNF-α+SM group showed an increased oxidative intracellular milieu, similar to that observed in the TNF-α group.

The adhesion of circulating monocytes to endothelial cells is an important event causing vascular inflammation (Hopkins, 2013). As shown in Figure 2B, TNF-α treatment for 6 h caused a 3.04-fold increase in THP-1/HAECs adhesion compared to that in the control group. The TNF-α+LSM group showed significant attenuation of THP-1/HAECs adhesion (1.44-fold) compared to the control group. However, attenuation of THP-1/HAEC adhesion in the TNF-α+SM group was not significant compared to that in the TNF-α group.

TF initiates the coagulation cascade upon vascular injury. TF-specific procoagulant activity is induced in endothelial cells to initiate coagulation and thrombosis (Steffel et al., 2006). Compared to the control group, TNF-α treatment for 6 h increased TF activity by 2.26-fold, while the TNF-α+LSM group showed significantly attenuated TF activity (1.16-fold). Similar to the monocyte adhesion assay, attenuation of TF activity in the TNF-α+SM group was not significant compared to in the TNF-α group (Figure 2C).

### Evaluation of effects of LSM and SM on TNF-α-induced endothelial dysfunction-related gene and protein expression

**Figure 3:**
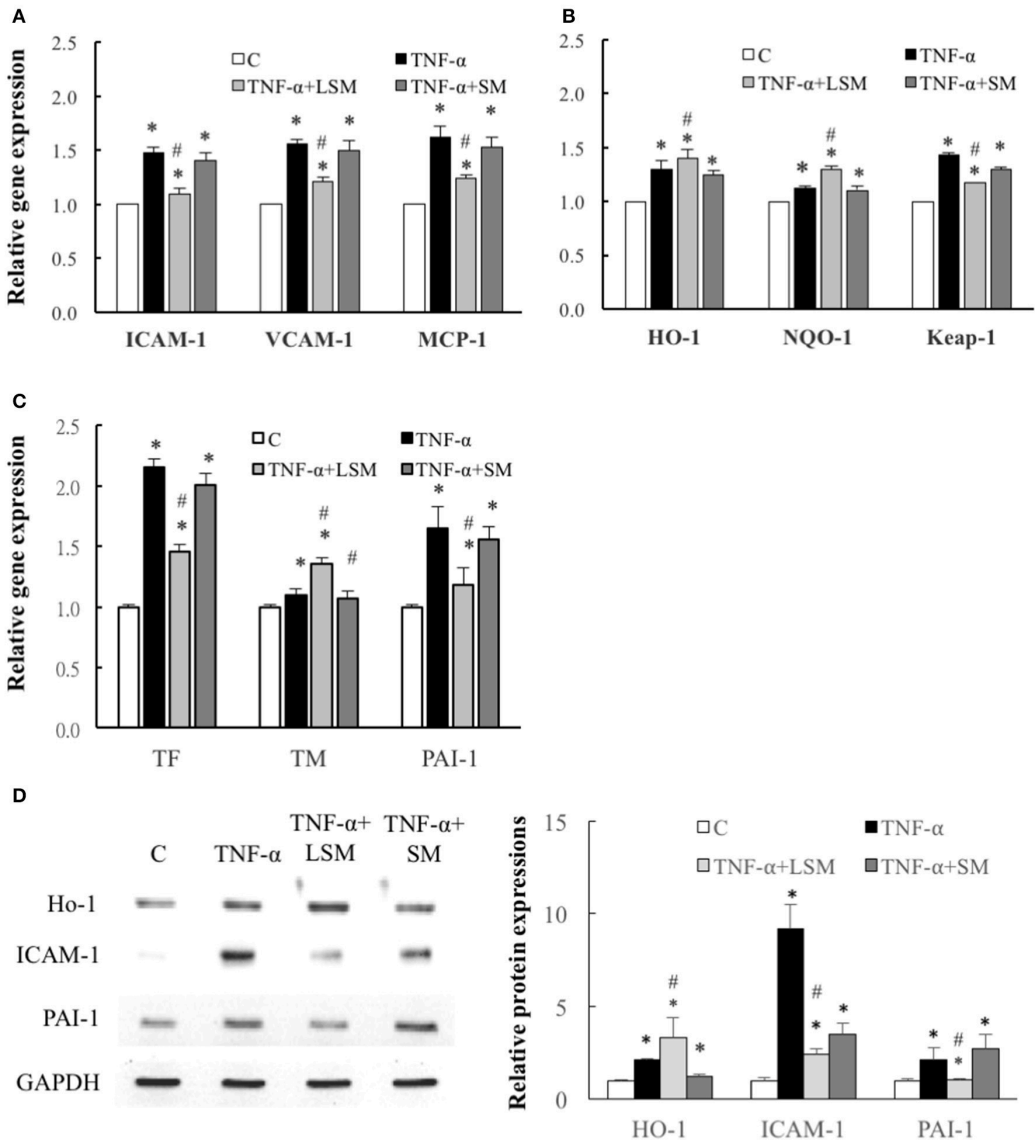
Determination of inflammation, ROS induction, and thrombosis-related gene and protein expression. Expression of **(A)** inflammation-related *ICAM-1, VCAM-1*, and *MCP-1* genes, **(B)** ROS induction–related *HO-1, NQO-1*, and *Keap-1* genes and **(C)** thrombosis-related *TF, TM*, and *PAI-1* genes were all significantly increased in the TNF-α group compared to in the control group. Gene expression levels of *ICAM-1, V-CAM-1, MCP-1, Keap-1, TF*, and *PAI-1* were attenuated significantly by LSM treatment compared to those in the TNF-α group, but similar trends were not observed in the TNF-α+SM group. However, gene expression of *HO-1, NQO-1*, and *TM* were significantly enhanced by LSM treatment compared to those in the TNF-α group, but similar trends were not observed in the TNF-α+SM group. **(A–C)** Data are expressed as the mean ± S.E.M. (*n* = 3). ^*^p < 0.05 indicates a significant difference relative to the control group. #*p* < 0.05 indicates a significant up- or downregulation, relative to the individual TNF-α group (excluding all control groups). **(D)** Compared with those in the control group, the protein levels of HO-1, ICAM-1, and PAI-1 were increased significantly in the TNF-α group. The expressions levels of the antioxidant protein HO-1 increased significantly in the TNF-α+LSM group compared to those in the TNF-α group. ICAM-1 and PAI-1 protein expression were attenuated significantly in the TNF-α+LSM group compared to those in the TNF-α group (*n* = 3). SM, static medium; LSM, new laminar shear medium. ^*^p < 0.05 indicates a significant difference relative to the control group. #*p* < 0.05 indicates a significant up- or downregulation relative to the individual TNF-α group.

Figure 3 shows that the gene expressions levels of inflammation-, thrombosis-, and ROS induction-related genes, *ICAM-1, VCAM-1, MCP-1, HO-1, NQO-1, Keap-1, TF, TM*, and *PAI-1* of the TNF-α group increased significantly by an average of 1.48-, 1.56-, 1.68-, 1.3-, 1.19-, 1.43-, 2.15-, 1.09-, and 1.65-fold, respectively, relative to the control group (Figures 3A–C). Compared to the TNF-α group, the expression levels of inflammation- and thrombosis-related genes, *ICAM-1, VCAM-1, MCP-1, TF*, and *PAI-1* in the TNF-α+LSM groups for 6 h were significantly attenuated (Figures 3A,C). Expression levels of the anti-thrombotic TM gene increased significantly by an average of 1.25-fold (1.36/1.09) relative to the TNF-α group (Figure 3C). Among the three ROS induction-related genes, *Keap-1* gene expression was significantly attenuated in the TNF-α+LSM groups. The expression levels of the other *HO-1* and *NQO-1* antioxidant genes increased significantly by an average of 1.08-fold (1.4/1.3) and 1.09-fold (1.3/1.19) relative to the TNF-α group (Figure 3B). However, gene expression in the TNF-α+SM groups was not significantly different from that in the TNF-α group.

Western blotting revealed that the protein levels of HO-1, ICAM-1, and PAI-1 increased significantly in the TNF-α groups compared to in the control group. Compared to the TNF-α group, the expressions level of the antioxidant protein HO-1 increased significantly in the TNF-α+LSM group, but did not show the same trend in the TNF-α+SM group. ICAM-1 and PAI-1 expression was significantly attenuated in the TNF-α+LSM group compared to those in the TNF-α+SM group (Figure 3D). The data indicate consistent trends in gene and protein expression.

### Quantification of cytokines secreted by HAECs in SM and LSM by cytokine antibody array

**Figure 4:**
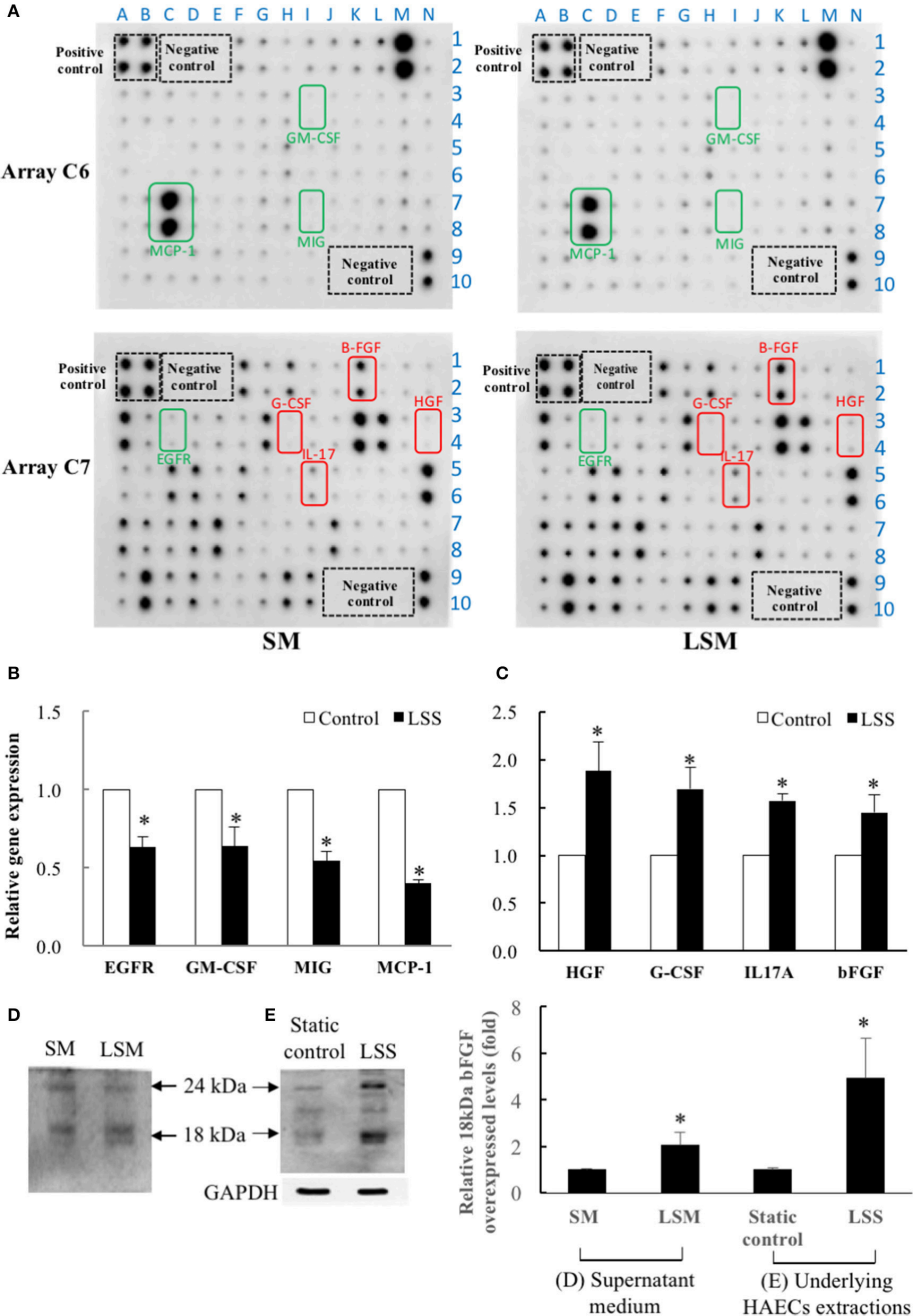
Quantification of HAEC-secreted cytokines in SM from static control and LSM from new LSS device by Human Cytokine Antibody Array C1000. **(A)** Left panels show SM data and right panels show LSM data. Array C6 is shown in upper two panels and array C7 is shown in bottom two panels. Among the 120 candidate cytokines, HAEC-secreted MCP-1 (C6) and bFGF (C7) were the two most abundant (O.D. level of each spot >5,000) and were accompanied by a maximum fold-change as the top under and over-expressed biomarkers. Secreted MCP-1 and bFGF proteins were 0.75- and 1.72-fold higher in LSM than in SM, respectively. The top three over-expressed marker cytokines in LSM are framed in red (HGF, G-CSF, and IL-17A), and the top three under-expressed cytokines (EGFR, GM-CSF, and MIG) in LSM are framed in green. A–N is X-axis and 1–10 is Y-axis, the XY combination indicated the location of each cytokine (Supplementary Data 3). **(B,C)** Comparison of gene expression levels of the above 8 marker cytokines in underlying HAECs between static control cells and cells exposed to LSS for 24 h by qPCR. Data are expressed as the mean ± S.E.M. (*n* = 3). ^*^p < 0.05 indicates a significant difference relative to the static control groups. **(D)** The expressions levels of the secreted autocrine type, 18-kDa bFGF protein levels, increased significantly in the LSM compared to in the SM. (*n* = 3) **(E)** Expressions levels of the 18-kDa bFGF protein levels increased significantly in HAECs exposed to LSS for 24 h compared to the static control. (*n* = 3). ^*^p < 0.05 indicates a significant difference relative to the SM **(D)** and static control **(E)** groups, respectively.

In Figure 4A, the left and right panels show SM and LSM data, respectively, and the array C6 and C7 are shown in upper and bottom panels, respectively. We quantified the raw numerical densitometry data and showed relative changes in the level of each cytokine in Supplementary Data 3. The top three over-expressed marker cytokines of LSM were HGF, G-CSF, and IL-17 (labeled in red), the top three under-expressed marker cytokines were GM-CSF, EGFR, and MIG (labeled in green), and the cytokine-spots are shown in the same color. Considering the dosage effects, we screened highly abundant marker cytokines (each densitometry data >5,000 in both the SM and LSM) from advanced experiments. Among all high-abundance marker cytokines, secreted bFGF (1.72-fold, LSM relative to SM) and MCP-1 (0.75-fold, LSM relative to SM) were the two most abundant, showing a maximum fold-change as the most under and over-expressed biomarkers, respectively (in bold type in Supplementary Data 3). Thus, we investigated whether bFGF protects the LSM from endothelial dysfunction. To confirm whether the above over-expressed and under-expressed cytokines were secreted from LSS-exposed HAECs, we collected the cell pellets to determine the mRNA transcript levels by qPCR. The data indicated that the gene levels in the underlying HAECs exposed to LSS for 24 h were as follows: under-expressed cytokines (EGFR, GM-CSF, MIG, and MCP-1) were 0.63-, 0.64-, 0.54-, and 0.4-fold lower than the static control (Figure 4B) and over-expressed cytokines (HGF, G-CSF, IL-17, and bFGF) were 1.89-, 1.7-, 1.57-, and 1.45-fold higher than the static control (Figure 4C). These trends are consistent with those of cytokine expression between SM and LSM, as shown in Figure 4A. In western blotting, the secreted autocrine type 18-kDa bFGF protein was not only over-expressed in LSM compared to in SM (Figure 4D), but also over-expressed in the underlying HAECs exposed to LSS for 24 h compared to in the static control (Figure 4E).

### Evaluation of protective efficacy of LSM, rbFGF, and LSM+Ab on TNF-α-induced endothelial ROS induction, inflammation, and thrombosis

**Figure 5:**
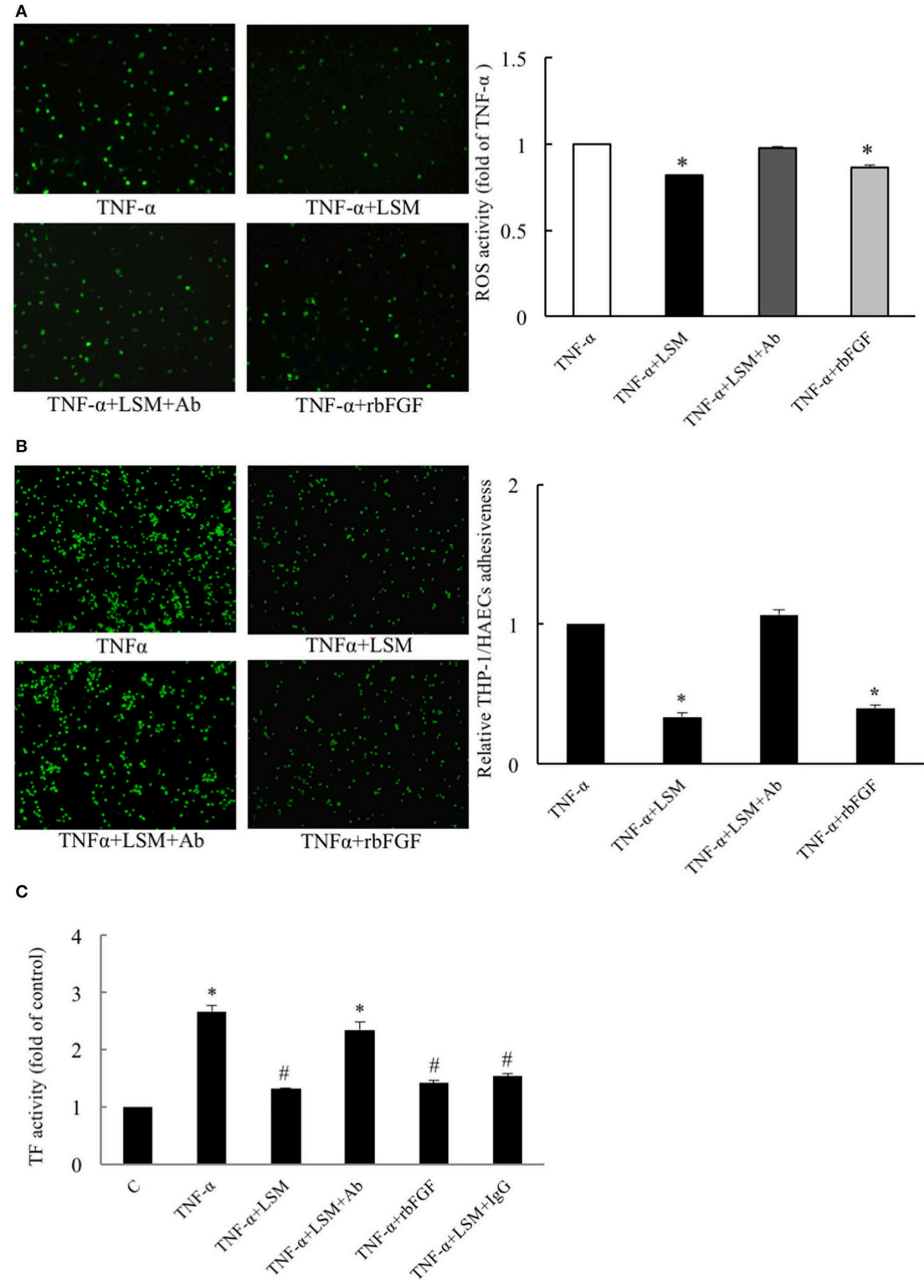
Evaluation of protective efficacies of LSM, rbFGF, and LSM+Ab on TNF-α-induced ROS activity, inflammation, and thrombosis. **(A)** The TNF-α group displayed a high level of H2DCFDA-dependent ROS activity, while the TNF-α+LSM and TNF-α+rbFGF groups showed significantly attenuated levels (average 0.81- and 0.86-fold) compared to the TNF-α group. **(B)** Compared to the TNF-α group, the high THP-1/HAECs adhesion ratios were attenuated significantly (average 0.34- and 0.4-fold) in the TNF-α+LSM and TNF-α+rbFGF groups. **(A,B)** The data are expressed as the mean ± S.E.M. (*n* = 4). ^*^p < 0.05 indicates significant downregulation relative to the TNF-α group. **(C)** Compared to the control group, the highest level of TF activity was increased by 2.66-fold in the TNF-α group. TF activity decreased significantly (average 1.32- and 1.42-fold) in the TNF-α+LSM and TNF-α+rbFGF group compared to the TNF-α group, respectively. However, the TNF-α+LSM+Ab group (average 2.35-fold) was not significantly different from the TNF-α group. The data are expressed as the mean ± S.E.M. (*n* = 4). ^*^p < 0.05 and #*p* < 0.05 indicate a significant difference relative to the control group and TNF-α group, respectively.

To validate the HAEC-secreted autocrine rbFGF as a critical protector in the LSM, we evaluated the protective efficacy of rbFGF and LSM+Ab using the H_2_DCFDA assay for ROS activity. As shown in Figure 5A, the TNF-α group displayed high levels of H_2_DCFDA-dependent fluorescence, while the TNF-α+LSM group showed significantly attenuated levels of H_2_DCFDA-dependent fluorescence (average 0.82-fold) compared to the TNF-α group. The TNF-α+rbFGF group also showed significantly attenuated levels of H_2_DCFDA-dependent fluorescence (average 0.87-fold) compared to the TNF-α group. The TNF-α+LSM+Ab group displayed ROS activity similar to that of the TNF-α group (average 0.98-fold). As shown in Figure 5B, the TNF-α+LSM and TNF-α+rbFGF groups showed significantly reduced adhesiveness of THP-1/HAECs (0.34- and 0.4-fold) compared to the TNF-α group. The adhesiveness of THP-1/HAECs was not changed in the TNF-α+LSM+Ab group compared to that in the TNF-α group. As shown in Figure 5C, only TNF-α treatment for 6 h revealed a 2.66-fold increase in TF activity compared to that in the control group. The TNF-α+LSM and TNF-α+rbFGF groups showed significantly decreased TF activity by 1.32- and 1.42-fold compared to the control TF activity level. Similar to in the THP-1/HAECs adhesion assay, the TNF-α+LSM+Ab group (2.35-fold) did not show a TF activity level that was significantly different from the level in the TNF-α group (Figure 5C).

### Evaluation of effects of LSM, rbFGF, LSM+Ab, and LSM+IgG on TNF-α-induced endothelial dysfunction-related gene and protein expression

**Figure 6:**
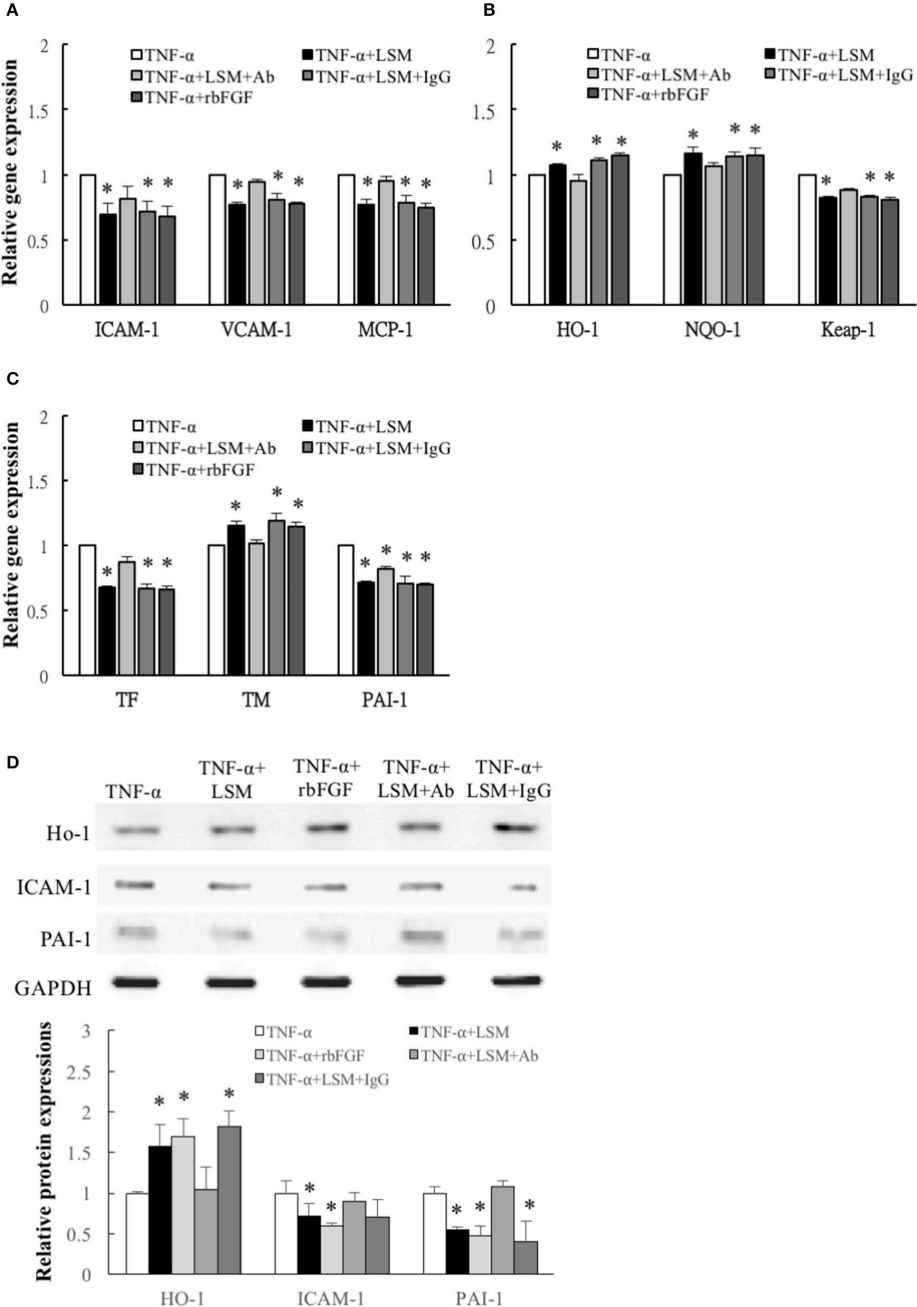
Determination of inflammation, ROS induction, and thrombosis-related gene and protein expression. **(A)** Inflammation related *ICAM-1, VCAM-1*, and *MCP-1* genes, **(B)** ROS induction related *Keap-1* genes, and **(C)** thrombosis-related TF and PAI-1 genes were attenuated significantly in the TNF-α+LSM, TNF-α+LSM+IgG, and TNF-α+rbFGF groups compared to in the TNF-α groups. Significant attenuation of *ICAM-1, VCAM-1, MCP-1, TF*, and *Keap-1* were not observed in the TNF-α+LSM+Ab groups. Additionally, ROS induction-related antioxidant HO-1, NQO-1 and anti-thrombotic TM gene levels were enhanced in the TNF-α+LSM, TNF-α+LSM+IgG, and TNF-α+rbFGF groups compared to in the TNF-α groups. **(A–C)** Data are expressed as the mean ± S.E.M. (*n* = 3). ^*^p < 0.05 indicates a significant difference relative to the TNF-α treated groups. **(D)** Paralleling their gene expression levels, the expression levels of ICAM-1 and PAI-1 protein decreased in the TNF-α+LSM, TNF-α+rbFGF, and TNF-α+LSM+IgG groups compared to in the TNF-α group. In addition, the expression levels of HO-1 protein increased in the TNF-α+LSM, TNF-α+rbFGF, and TNF-α+LSM+IgG groups compared to in the TNF-α group (*n* = 3). ^*^p < 0.05 indicates a significant different relative to the TNF-α group.

Compared with the TNF-α group, the expression levels of inflammation, thrombosis, and ROS induction-related genes, *ICAM-1, VCAM-1, MCP-1, HO-1, NQO-1, Keap-1, TF, TM*, and *PAI-1* in the TNF-α+LSM, TNF-α+LSM+IgG, and TNF-α+rbFGF groups differed significantly (Figures 6A–C). However, *ICAM-1, VCAM-1, MCP-1, HO-1, NQO-1, Keap-1, TF*, and *TM* gene expression levels in the TNF-α+LSM+Ab group did not significantly differ from those in the TNF-α group (Figures 6A–C). Unexpectedly, only *PAI-1* gene expression levels in the TNF-α+LSM+Ab group decreased significantly compared to the TNF-α group. Western blotting revealed that the protein levels of ICAM-1 and PAI-1 were attenuated in the TNF-α+LSM, TNF-α+rbFGF, and TNF-α+LSM+IgG groups compared to that in the TNF-α group (Figure 6D). In contrast, protein expression levels of HO-1 increased significantly in the TNF-α+LSM, TNF-α+rbFGF, and TNF-α+LSM+IgG groups compared to that in the TNF-α group. These data indicate that the pattern of gene and protein expression was nearly consistent in this study.

### Evaluation of effects of LSM, rbFGF, and LSM+Ab on acute TNF-α-induced endothelial dysfunction and lung injury in C57BL/6J mice

**Figure 7:**
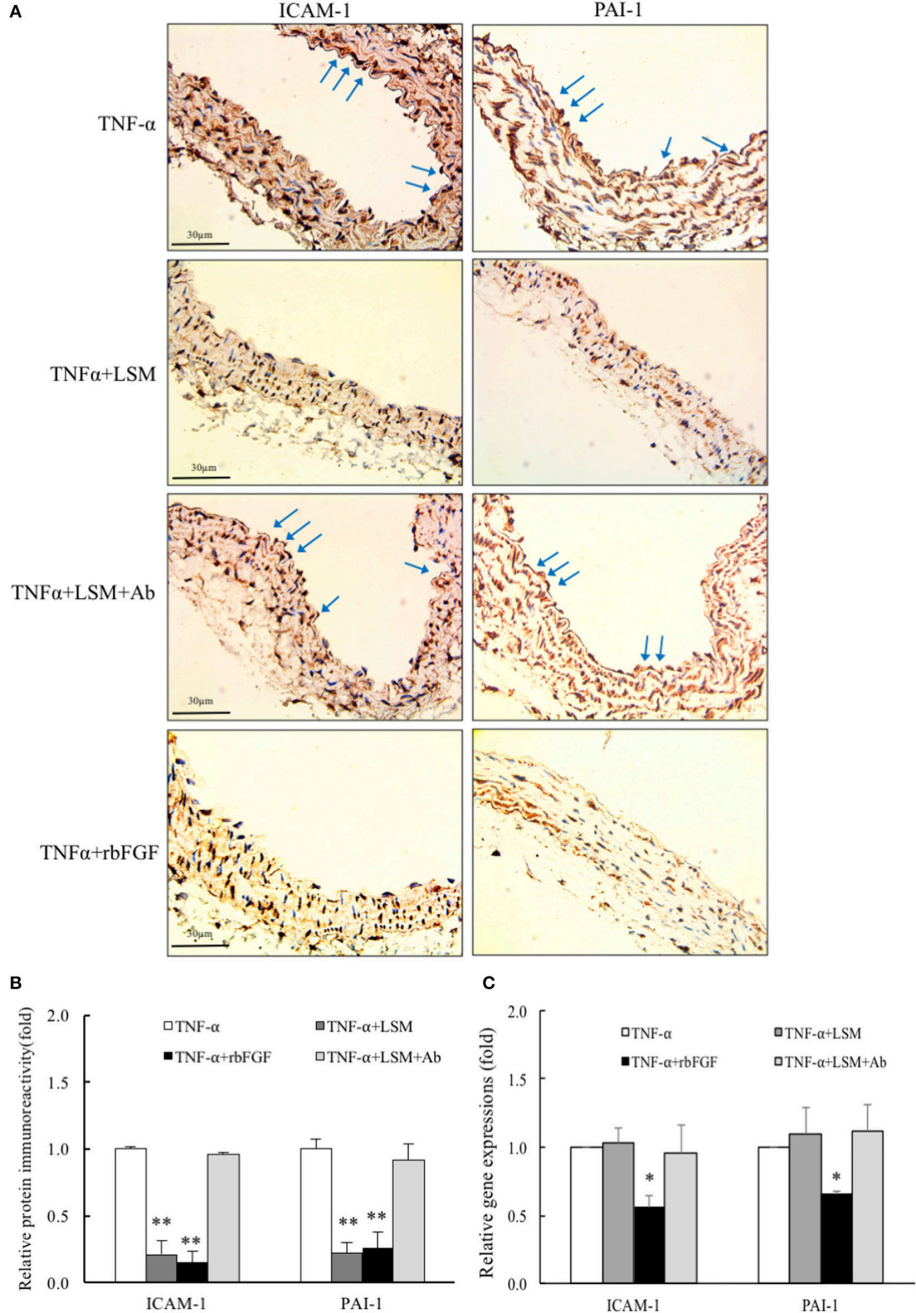
LSM and rbFGF suppresses TNF-α-induced ICAM-1 and PAI-1 protein and gene expression *in vivo*. **(A)** Immunohistochemical staining of ICAM-1 and PAI-1 proteins in thoracic aorta tissue. Representative images showing immunoreactivities of ICAM-1 and PAI-1 in the aortic endothelial layers (brown color as arrow) were decreased in the TNF-α+LSM and TNF-α+rbFGF groups compared to in the TNF-α group. The immunoreactivities of ICAM-1 and PAI-1 in the aortic endothelial layer were not decreased in the TNF-α+LSM+Ab group compared to in the TNF-α group (*n* = 3). **(B)** Immunoreactivity signals in endothelial layers from 4 groups was quantified for all immunohistochemical images. ^**^p < 0.01 indicates a significant difference relative to the TNF-α group. **(C)** At 24 h after TNF-α i.p. injection, *ICAM-1* and *PAI-1* gene expressions were reduced by approximately to 0.55- and 0.64-fold in lung tissues in the TNF-α+rbFGF groups compared to in the TNF-α group. Data are expressed as the mean ± S.E.M. (*n* = 3). ^*^p < 0.05 indicates a significant difference relative to the TNF-α group.

As shown in Figure 7A, immunohistochemical analysis showed that aortic endothelial ICAM-1 and PAI-1 immunoreactivity levels in the TNF-α+LSM and TNF-α+rbFGF groups were decreased dramatically compared to in the TNF-α and TNF-α+LSM+Ab groups. No immunoreactivity was detected when the primary antibody was omitted (data not shown). Quantification of immunoreactivity signals in the endothelial layers revealed that ICAM-1 expression was decreased significantly to 0.21- and 0.15-fold in the TNF-α+LSM and TNF-α+rbFGF groups compared to that in the TNF-α group. PAI-1 expression in the endothelial layers decreased significantly to 0.22- and 0.26-fold in the TNF-α+LSM and TNF-α+rbFGF groups compared to in the TNF-α group (Figure 7B). The immunoreactivity of these two proteins did not differ significantly between the TNF-α group and TNF-α+LSM+Ab group. In addition, 24 h after TNF-α injection, ICAM-1 and PAI-1 mRNA expression was significantly reduced by approximately to 0.55- and 0.64-fold from that in the TNF-α group in lung extractions by co-injection of rbFGF, respectively. Unexpectedly, the dramatic reductions in ICAM-1 and PAI-1 mRNA expression were not observed in the TNF-α+LSM group (Figure 7C). Interestingly, the highest serum bFGF levels were found in the TNF-α+rbFGF group (Supplementary Data 4) and the serum levels of bFGF increased by injection of rbFGF correlated with positive effects on lung tissues. These results suggest that intraperitoneal injection of rbFGF has more protective potential as a therapeutic drug than LSM for inhibiting inflammation and thrombosis in TNF-α-induced tissue injury by reversing the increased expression of ICAM-1 and PAI-1.

### rbFGF inhibited inflammatory- and thrombosis-related gene and protein expression via activation of MEK5-KLF2 and p38-MAPK signal pathway

**Figure 8:**
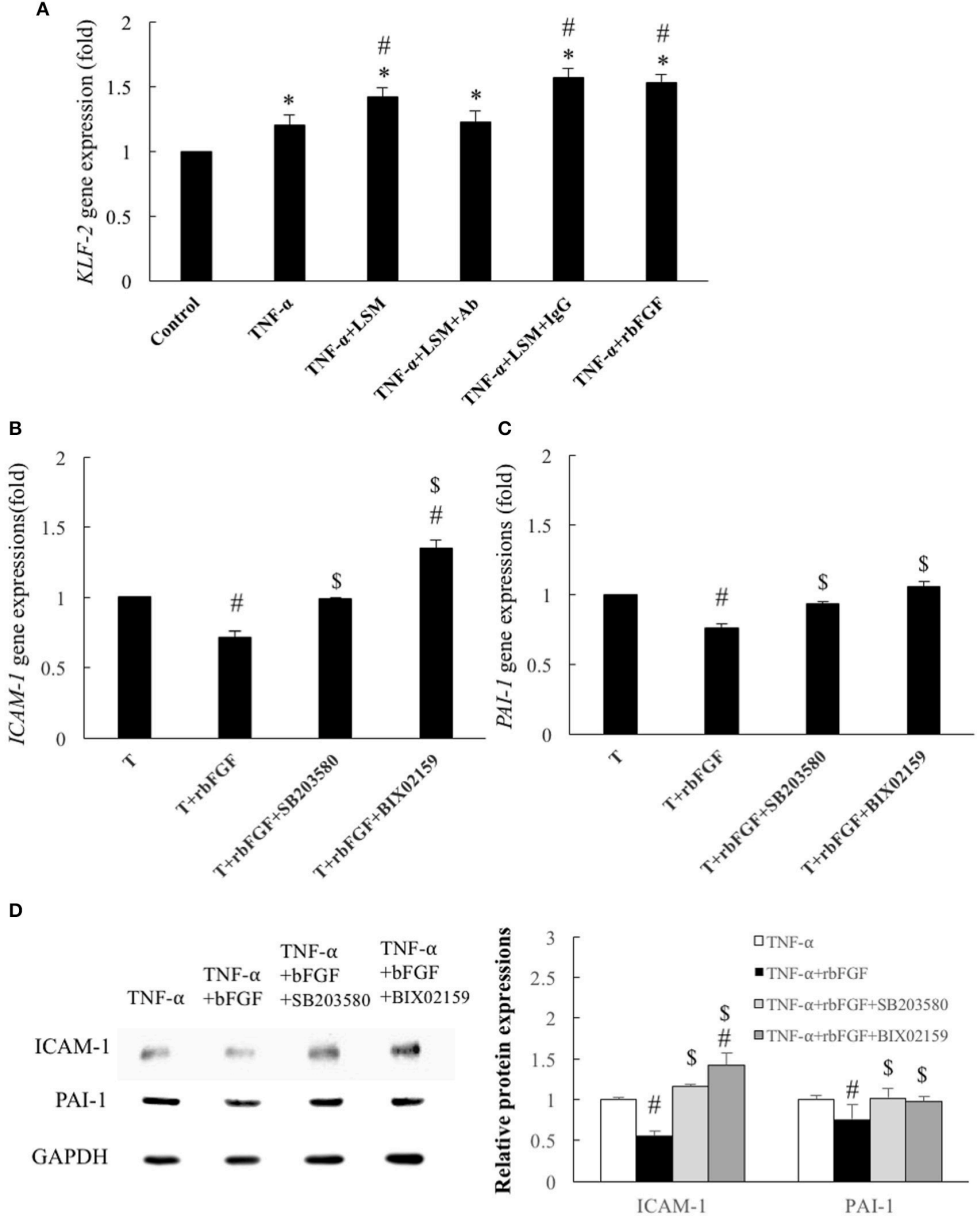
Identification of cytoprotective mechanisms of bFGF. **(A)** Gene expression of *KLF2* was increased significantly by 1.2-fold by TNF-α, compared to the control group. In the TNF-α+LSM, TNF-α+LSM+IgG, and TNF-α+rbFGF groups, the gene expression of *KLF2* was further enhanced by 1.41-, 1.57-, and 1.53- fold compared to the control groups. The KLF2 level in the TNF-α+LSM+Ab group was not significantly different from that in the TNF-α group. Data are expressed as the mean ± S.E.M. (*n* = 4). ^*^p < 0.05 and #*p* < 0.05 indicate a significant different relative to the control group and TNF-α group, respectively. **(B)** Compared to the TNF-α group, *ICAM-1* expression was significantly decreased to 0.71-fold in the TNF-α+bFGF group. However, the anti-inflammatory effect of bFGF on *ICAM-1* expression was reversed to 0.99- and 1.35-fold compared to in the TNF-α group by pretreatment with SB203580 (10 μM) and BIX02189 (10 μM), respectively. **(C)** Compared to the TNF-α group, the *PAI-1* expression level was significantly decreased to 0.76-fold in the TNF-α+bFGF group. However, the anti-thrombotic effect of bFGF on PAI-1 gene expression was reversed to 0.93- and 1.05-fold compared to in the TNF-α group by pretreatment with SB203580 and BIX02189, respectively. T: TNF-α **(B,C)** Data are expressed as the mean ± S.E.M. (*n* = 4). #*p* < 0.05 indicates a significant difference relative to the TNF-α group and $ *p* < 0.05 indicates a significant difference relative to the TNF-α+rbFGF group. **(D)** Compared with the TNF-α group, ICAM-1 and PAI-1 protein levels were significantly decreased in the TNF-α+rbFGF group and were reverted by pretreatment with SB203580 and BIX02189, respectively (*n* = 3). #*p* < 0.05 indicates a significant difference relative to the TNF-α group and $ *p* < 0.05 indicates a significant difference relative to the TNF-α+rbFGF group.

**Figure 9:**
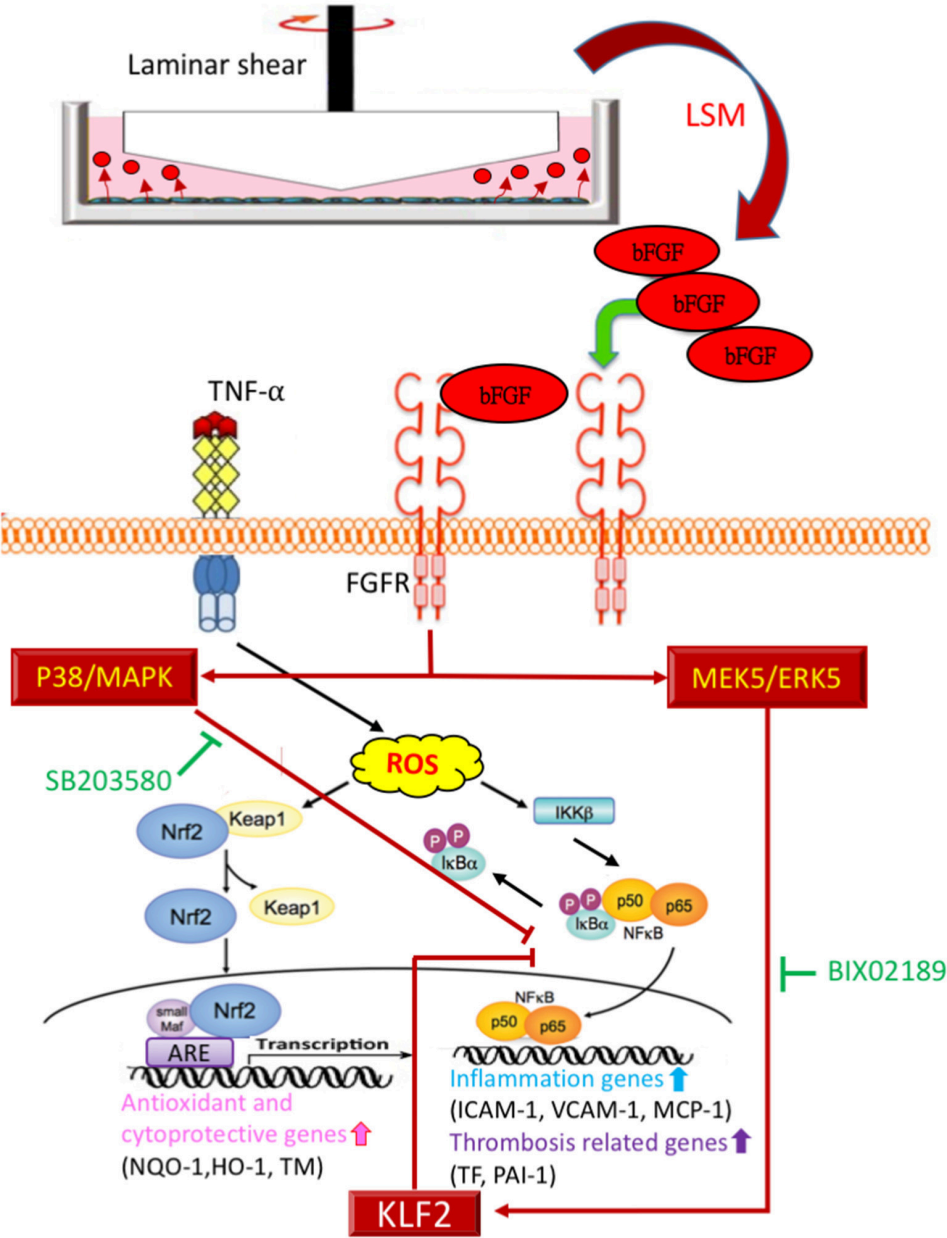
Proposed model of cytoprotective mechanism of bFGF in TNF-α stimulated endothelial dysfunction via p38-MAPK and MEK5/ERK5-KLF2 pathways.

Following the above *in vivo* study, we evaluated the protective mechanism of rbFGF. We examined whether the protective mechanism of bFGF also depended on the laminar shear-responsive transcription factor, KLF2, as the classical atheroprotection pathway of LSS (Dekker et al., 2002; Fledderus et al., 2008). As shown in Figure 8A, rbFGF did induce significant overexpression of the KLF2 gene compared to in the control and TNF-α groups and achieved similar expression levels as the KLF2 gene in the TNF-α+LSM and TNF-α+LSM+IgG groups. Moreover, we used BIX02159 (specific inhibitor of MEK5) and SB203580 (specific inhibitor of p38-MAPK) to test the protective mechanism of rbFGF in TNF-α-induced ICAM-1 and PAI-1 gene overexpression. As shown in Figures 8B–D, pretreatment with SB203580 and BIX02189 reversed the inhibition of ICAM-1 and PAI-1 gene and protein overexpression from the TNF-α+bFGF groups. The inhibition assay revealed that the protective mechanism of bFGF on inflammation and thrombosis involved activation of the p38-MAPK and MEK5/ERK5-KLF2 signal pathway to inhibit nuclear factor-κB downstream target genes, *ICAM1-1* and *PAI-1*, following TNF-α stimulation (Figure 9).

## Discussion

In our modified cone-and-plate system, HAECs were seeded and exposed to LSS for 24 h. In the peripheral area of the 10-cm culture dish, HAECs exposed to LSS exhibited the typical alignment of cell shape in the direction of the laminar flow. In addition, expression levels of mechanosensitive genes of the underlying HAECs, TM, HO-1, NQO-1, and KLF-2, were also upregulated, similar to the results observed for the traditional LSS system. The MTS assay confirmed the non-toxicity of the new LSM. These data validated that our modified system also benefits the underlying HAECs, similar to the traditional cone-and-plate system. Moreover, we showed that the new LSM of the modified system was more effective than the traditional system in preventing TNF-α-induced endothelial dysfunction based on qPCR of ICAM-1, HO-1, and PAI-1 gene levels. We also demonstrated that the LSM of the new LSS system has therapeutic potential to rescue TNF-α-induced dysfunction in HAECs, based on both quantitative and qualitative analyses. To our knowledge, this is the first study to report the application of a modified traditional cone-and-plate shear system in studies investigating LSS-mediated paracrine and autocrine effects.

In 2004, Chiu et al. showed that high shear stress (20 dynes/cm^2^) enhanced the TNF-α-induced expression of ICAM-1 at the mRNA and surface-protein levels in underlying HUVECs, but suppressed TNF-α-induced expression of VCAM-1 and E-selectin proteins (Chiu et al., 2004). In 2015, Fan et al. also demonstrated that both high shear stress and oscillatory shear stress induced over-expression of ICAM-1 in the underlying HUVECs relative to the levels in the static control, although ICAM-1 levels were lower under higher shear stress (Fan et al., 2015). Interestingly, our data showed that LSM could overcome TNF-α-induced ICAM-1 gene and protein overexpression in the underlying HAECs. A possible explanation is that increased ICAM-1 levels have only been observed in venous endothelial cells, HUVECs. Thus, studies of aortic endothelial cells, HAECs, may show different results. Moreover, the increased ICAM-1 levels in underlying HUVECs may be induced by direct physical shear stress. Instead, the new LSM contained cytoprotective substances that functioned in an autocrine and paracrine manner by decreasing TNF-α-induced ICAM-levels in HAECs.

In the disturbed flow, Urschel et al. reported that HUVECs exposed to flow in bifurcating slides secreted increased levels of ICAM-1 and interleukin-8 in conditioned medium compared to cells grown under laminar flow (Urschel et al., 2012). Additionally, Bajari et al. reported that conditioned medium from chronic low shear stress (2.5 dynes/cm^2^) enhanced the migration of vascular smooth muscle cells (Bajari et al., 2014). In 2015, Franzoni et al. also reported that conditioned medium from endothelial cells exposed to reciprocating flows could increase the proliferation of smooth muscle cells (Franzoni et al., 2016). In contrast, under laminar flow, Slater et al. reported that conditioned medium from glomerular endothelial cells under chronic LSS (10 dynes/cm^2^) decreased podocyte monolayer resistance (Slater et al., 2012). These reports suggest the potential bio-functional flow-mediated effects of “conditioned media” when shear stress is applied to the underlying endothelial cells. Using the modified cone-and-plate shear device, we also found that LSM was effective in rescuing cells from TNF-α-induced endothelial dysfunction.

Previous studies indicated that upon exposure to environmental signals, cytokines in vascular endothelial cells undergo changes in gene expression and function that allow these cells to actively participate in inflammatory reactions, immunity, and thrombosis (Mantovani et al., 1992). In this study, the same commercial medium was used in both the static control and LSS system and the underlying HAECs did secrete diverse cytokines into LSM to provide significant protective effects compared to SM (Figure 4). Considering the effect of dosage, we isolated highly abundant cytokines (all OD > 5,000) as marker cytokines, and sorted all marker cytokines by relative changes in levels in the LSM and SM. Thus, the most over-expressed bFGF (LSM/SM is 1.72-fold) was chosen as the target marker cytokine in this study (Supplementary Data 3). Based on *in vitro* and *in vivo* experiments, bFGF was identified as a critical protective factor in LSM against endothelial dysfunction (Figures 5–8).

The FGF family is comprised secreted signaling proteins (secreted FGFs) that signal to receptor tyrosine kinases and intracellular non-signaling proteins (intracellular FGFs) (Ornitz and Itoh, 2015). bFGF (*FGF-2*) is the prototype member of a family of heparin-binding growth factors, and lacks a signal peptide that directs secretion through the classical secretory pathway (Bikfalvi et al., 1997; Ornitz and Itoh, 2015). It is also a multifunctional protein, translated from the same mRNA as its high molecular weight (21–24 kDa; Hi-bFGF) or low molecular weight (18 kDa; Lo-bFGF) isoforms. Hi-bFGF localizes preferentially to the cell nucleus and exerts exclusively intracrine activities. In contrast, autocrine or paracrine bFGF activities were demonstrated to represent the action of Lo-bFGF, also known as secreted-bFGF (Kardami et al., 2004). A previous study suggested that endothelial cell-derived bFGF mediates angiogenesis in an autocrine manner in cancer (Seghezzi et al., 1998). This is the first study to demonstrate that LSS-induced aortic endothelial cells over-expressed and secreted autocrine type 18 kDa bFGF into the external environment (Figures 4C,D). In cultured microvascular endothelial cells, bFGF induced the development of an angiogenic phenotype including increased proliferation, migration, and proteinase production (Bikfalvi et al., 1997; Seghezzi et al., 1998). In addition, in cancer research, bFGF signaling was showed to possess powerful cardio-protective effects following stress and ischemic injury (Kardami et al., 2004; House et al., 2005; Liao et al., 2007). Mice lacking the bFGF gene develop normally, but show reduced vascular tone, impaired cardiac hypertrophy, reduced cortical neuron density, and defects in response to pulmonary or cardiac injury (Dono et al., 2002; House et al., 2010). No previous studies have shown that secreted bFGF from LLS-exposed HAECs can prevent endothelial dysfunction. In this study, we evaluated the protective efficacy of rbFGF and LSM+Ab (bFGF neutralization by monoclonal Ab) against TNF-α-induced HAEC dysfunction *in vitro* and *in vivo* (Figures 5–7). The results suggest significant protective effects of rbFGF (10 ng/mL), similar to LSM against TNF-α-induced endothelial dysfunction. Interestingly, the protective effects of LSM were only observed in aortic endothelial dysfunction, but not reproduced in lung extractions *in vivo* (Figure 7B). These results suggest that bFGF has more protective effects than LSM against inflammation and thrombosis following TNF-α stimulation *in vivo*.

Atheroprotective blood flow induces the expression of anti-inflammatory KLF2 expression, a transcriptional factor responsible for the physiological healthy, flow-exposed state of endothelial cells (Fledderus et al., 2008; Chiu and Chien, 2011). In a previous study, Parmar et al. documented that KLF2 is induced under laminar flow via MAPK/ERK kinase 5 (MEK5)-extracellular signal-regulated protein kinase 5 (ERK5)-MEF2 signaling pathway (Parmar et al., 2006). In addition, Flati et al. reported that bFGF can inhibit the activation of nuclear factor-κB and ICAM-1 elevation by inducing p38-MAPK (Flati et al., 2006). Thus, we tested KLF2 expression among the different treated groups and used the specific inhibitors BIX02159 (MEK5 inhibitor) and SB203580 (P38 inhibitor) to evaluate the individual reversions of anti-inflammatory and anti-thrombotic protections of bFGF. Similar to previous KLF2 and bFGF associated studies, we found that pretreatment with SB203580 and BIX02189 inhibitors significantly reversed the protection of bFGF on KLF2 downstream ICAM-1 and PAI-1 gene and protein reductions from the TNF-α+bFGF groups (Figures 8B–D).

This study implies three novel findings: (1) LSM collected from the new LSS system had more protective effects against TNF-α stimulated endothelial dysfunction, than traditional LSM. (2) LSS-exposed HAEC-secreted autocrine bFGF is the critical factor in the LSM that provides diverse cytoprotective effects against TNF-α-stimulated endothelial dysfunction. (3) The protective mechanism of bFGF via activation of the p38-MAPK and MEK5/ERK5-KLF2 signal pathway inhibits nuclear factor-κB downstream target genes (Figure 9). Based on previously reported variable atheroprotective effects of LSS and our *in vitro* and *in vivo* results, autocrine-type secreted bFGF is a critical protector in LSM, providing a new perspective for vascular endothelial shear stress studies.

## Author contributions

H-JW and W-YL designed and performed the experiments; analyzed the data and wrote the manuscript; provided the funding. W-YL supervised the study.

### Conflict of interest statement

The authors declare that the research was conducted in the absence of any commercial or financial relationships that could be construed as a potential conflict of interest.

## References

[B1] Bajari T. M. Winnicki W. Gensberger E. T. Scharrer S. I. Regele H. Aumayr K.. (2014). Known players, new interplay in atherogenesis: chronic shear stress and carbamylated-LDL induce and modulate expression of atherogenic LR11 in human coronary artery endothelium. Thromb. Haemost. 111, 323–332. 10.1160/TH12-12-0924 24284991

[B2] Bikfalvi A. Klein S. Pintucci G. Rifkin D. B. (1997). Biological roles of fibroblast growth factor-2. Endocr. Rev. 18, 26–45. 10.1210/er.18.1.26 9034785

[B3] Blake G. J. Ridker P. M. (2002). Tumour necrosis factor-alpha, inflammatory biomarkers, and atherogenesis. Eur. Heart J. 23, 345–347. 10.1053/euhj.2001.2905 11846489

[B4] Branen L. Hovgaard L. Nitulescu M. Bengtsson E. Nilsson J. Jovinge S. (2004). Inhibition of tumor necrosis factor-alpha reduces atherosclerosis in apolipoprotein E knockout mice. Arterioscler. Thromb. Vasc. Biol. 24, 2137–2142. 10.1161/01.ATV.0000143933.20616.1b 15345516

[B5] Chen B. Lu Y. Chen Y. Cheng J. (2015). The role of Nrf2 in oxidative stress-induced endothelial injuries. J. Endocrinol. 225, R83–R99. 10.1530/JOE-14-0662 25918130

[B6] Chen B. P. Li Y. S. Zhao Y. Chen K. D. Li S. Lao J.. (2001). DNA microarray analysis of gene expression in endothelial cells in response to 24-h shear stress. Physiol. Genomics 7, 55–63. 10.1006/geno.2001.6511 11595792

[B7] Chien S. (2007). Mechanotransduction and endothelial cell homeostasis: the wisdom of the cell. Am. J. Physiol. 292, H1209–H1224. 10.1152/ajpheart.01047.2006 17098825

[B8] Chiu J. J. Chien S. (2011). Effects of disturbed flow on vascular endothelium: pathophysiological basis and clinical perspectives. Physiol. Rev. 91, 327–387. 10.1152/physrev.00047.2009 21248169 PMC3844671

[B9] Chiu J. J. Lee P. L. Chen C. N. Lee C. I. Chang S. F. Chen L. J.. (2004). Shear stress increases ICAM-1 and decreases VCAM-1 and E-selectin expressions induced by tumor necrosis factor-[alpha] in endothelial cells. Arterioscler. Thromb. Vasc. Biol. 24, 73–79. 10.1161/01.ATV.0000106321.63667.24 14615388

[B10] Dekker R. J. van Soest S. Fontijn R. D. Salamanca S. de Groot P. G. VanBavel E.. (2002). Prolonged fluid shear stress induces a distinct set of endothelial cell genes, most specifically lung Kruppel-like factor (KLF2). Blood 100, 1689–1698. 10.1182/blood-2002-01-0046 12176889

[B11] Dono R. Faulhaber J. Galli A. Zuniga A. Volk T. Texido G.. (2002). FGF2 signaling is required for the development of neuronal circuits regulating blood pressure. Circ. Res. 90, E5–E10. 10.1161/hh0102.103611 11786528

[B12] Fan W. Fang R. Wu X. Liu J. Feng M. Dai G.. (2015). Shear-sensitive microRNA-34a modulates flow-dependent regulation of endothelial inflammation. J. Cell Sci. 128, 70–80. 10.1242/jcs.154252 25395581

[B13] Federici M. Menghini R. Mauriello A. Hribal M. L. Ferrelli F. Lauro D.. (2002). Insulin-dependent activation of endothelial nitric oxide synthase is impaired by O-linked glycosylation modification of signaling proteins in human coronary endothelial cells. Circulation 106, 466–472. 10.1161/01.CIR.0000023043.02648.51 12135947

[B14] Firasat S. Hecker M. Binder L. Asif A. R. (2014). Advances in endothelial shear stress proteomics. Expert Rev. Proteomics 11, 611–619. 10.1586/14789450.2014.933673 25017810

[B15] Flati V. Pastore L. I. Griffioen A. W. Satijn S. Toniato E. D'Alimonte I.. (2006). Endothelial cell anergy is mediated by bFGF through the sustained activation of p38-MAPK and NF-κB inhibition. Int. J. Immunopathol. Pharmacol. 19, 761–773. 10.1177/039463200601900406 17166398

[B16] Fledderus J. O. Boon R. A. Volger O. L. Hurttila H. Ylä-Herttuala S. Pannekoek H.. (2008). KLF2 primes the antioxidant transcription factor Nrf2 for activation in endothelial cells. Arterioscler. Thromb. Vasc. Biol. 28, 1339–1346. 10.1161/ATVBAHA.108.165811 18467642

[B17] Franzoni M. Cattaneo I. Longaretti L. Figliuzzi M. Ene-Iordache B. Remuzzi A.. (2016). Endothelial cell activation by hemodynamic shear stress derived from arteriovenous fistula for hemodialysis access. Am. J. Physiol. Heart Circ. Physiol. 310, H49–H59. 10.1152/ajpheart.00098.2015 26497959

[B18] Freed J. K. Greene A. S. (2010). Proteomic analysis of shear stress-mediated protection from TNF-α in endothelial cells. Microcirculation 17, 259–270. 10.1111/j.1549-8719.2010.00031.x 20536739 PMC3712086

[B19] Hopkins P. N. (2013). Molecular biology of atherosclerosis. Physiol. Rev. 93, 1317–1542. 10.1152/physrev.00004.2012 23899566

[B20] House S. L. Branch K. Newman G. Doetschman T. Schultz Jel J. (2005). Cardioprotection induced by cardiac-specific overexpression of fibroblast growth factor-2 is mediated by the MAPK cascade. Am. J. Physiol. 289, H2167–H2175. 10.1152/ajpheart.00392.2005 16040717

[B21] House S. L. House B. E. Glascock B. Kimball T. Nusayr E. Schultz J. E.. (2010). Fibroblast growth factor 2 mediates isoproterenol-induced cardiac hypertrophy through activation of the extracellular regulated kinase. Mol. Cell. Pharmacol. 2, 143–154. 10.4255/mcpharmacol.10.20 21274419 PMC3026329

[B22] Huang R. P. (2007). An array of possibilities in cancer research using cytokine antibody arrays. Expert Rev. Proteomics 4, 299–308. 10.1586/14789450.4.2.299 17425464

[B23] Kardami E. Jiang Z. S. Jimenez S. K. Hirst C. J. Sheikh F. Zahradka P.. (2004). Fibroblast growth factor 2 isoforms and cardiac hypertrophy. Cardiovasc. Res. 63, 458–466. 10.1016/j.cardiores.2004.04.024 15276471

[B24] Liao S. Porter D. Scott A. Newman G. Doetschman T. Schultz Jel J. (2007). The cardioprotective effect of the low molecular weight isoform of fibroblast growth factor-2: the role of JNK signaling. J. Mol. Cell. Cardiol. 42, 106–120. 10.1016/j.yjmcc.2006.10.005 17150229 PMC1852491

[B25] Lo W. Y. Peng C. T. Wang H. J. (2017). MicroRNA-146a-5p Mediates high glucose-induced endothelial inflammation via targeting Interleukin-1 receptor-associated Kinase 1 expression. Front. Physiol. 8:551. 10.3389/fphys.2017.00551 28824448 PMC5539227

[B26] Mantovani A. Bussolino F. Dejana E. (1992). Cytokine regulation of endothelial cell function. FASEB J. 6, 2591–2599. 1592209 10.1096/fasebj.6.8.1592209

[B27] Matsumoto Y. Kawai Y. Watanabe K. Sakai K. Murata M. Handa M.. (1998). Fluid shear stress attenuates tumor necrosis factor-alpha-induced tissue factor expression in cultured human endothelial cells. Blood 91, 4164–4172. 9596663

[B28] McCormick S. M. Eskin S. G. McIntire L. V. Teng C. L. Lu C.-M. Russell C. G.. (2001). DNA microarray reveals changes in gene expression of shear stressed human umbilical vein endothelial cells. Proc. Natl. Acad. Sci. U.S.A. 98, 8955–8960. 10.1073/pnas.171259298 11481467 PMC55355

[B29] Ornitz D. M. Itoh N. (2015). The fibroblast growth factor signaling pathway. Wiley Interdiscip. Rev. Dev. Biol. 4, 215–266. 10.1002/wdev.176 25772309 PMC4393358

[B30] Papaioannou T. G. Stefanadis C. (2005). Vascular wall shear stress: basic principles and methods. Hellenic J. Cardiol. 46, 9–15. 15807389

[B31] Parmar K. M. Larman H. B. Dai G. Zhang Y. Wang E. T. Moorthy S. N.. (2006). Integration of flow-dependent endothelial phenotypes by Kruppel-like factor 2. J. Clin. Invest. 116, 49–58. 10.1172/JCI24787 16341264 PMC1307560

[B32] Rezvan A. Ni C. W. Alberts-Grill N. Jo H. (2011). Animal, *in vitro*, and *ex vivo* models of flow-dependent atherosclerosis: role of oxidative stress. Antioxid. Redox Signal. 15, 1433–1448. 10.1089/ars.2010.3365 20712399 PMC3144429

[B33] Seghezzi G. Patel S. Ren C. J. Gualandris A. Doetschman T. Schultz J. E. J.. (1998). Fibroblast growth factor-2 (FGF-2) induces vascular endothelial growth factor (VEGF) expression in the endothelial cells of forming capillaries: an autocrine mechanism contributing to angiogenesis. J. Cell Biol. 141, 1659–1673. 10.1083/jcb.141.7.1659 9647657 PMC2132998

[B34] Shanmugam G. Narasimhan M. Sakthivel R. Kumar R. R. Davidson C. Palaniappan S.. (2016). A biphasic effect of TNF-alpha in regulation of the Keap1/Nrf2 pathway in cardiomyocytes. Redox Biol. 9, 77–89. 10.1016/j.redox.2016.06.004 27423013 PMC4961303

[B35] Slater S. C. Ramnath R. D. Uttridge K. Saleem M. A. Cahill P. A. Mathieson P. W.. (2012). Chronic exposure to laminar shear stress induces Kruppel-like factor 2 in glomerular endothelial cells and modulates interactions with co-cultured podocytes. Int. J. Biochem. Cell Biol. 44, 1482–1490. 10.1016/j.biocel.2012.05.020 22683691

[B36] Steffel J. Luscher T. F. Tanner F. C. (2006). Tissue factor in cardiovascular diseases: molecular mechanisms and clinical implications. Circulation 113, 722–731. 10.1161/CIRCULATIONAHA.105.567297 16461845

[B37] Tarbell J. M. Shi Z. D. Dunn J. Jo H. (2014). Fluid mechanics, arterial disease, and gene expression. Annu. Rev. Fluid Mech. 46, 591–614. 10.1146/annurev-fluid-010313-141309 25360054 PMC4211638

[B38] Urschel K. Cicha I. Daniel W. G. Garlichs C. D. (2012). Shear stress patterns affect the secreted chemokine profile in endothelial cells. Clin. Hemorheol. Microcirc. 50, 143–152. 10.3233/CH-2011-1450. 22538542

[B39] Wang H. J. Huang Y. L. Shih Y. Y. Wu H. Y. Peng C.-T. Lo W.-Y. (2014). MicroRNA-146a decreases high glucose/thrombin-induced endothelial inflammation by inhibiting NAPDH oxidase 4 expression. Mediators Inflamm. 2014:379537. 10.1155/2014/379537 25298619 PMC4179945

[B40] Wang H. J. Lo W. Y. Lu T. L. Huang H. (2010). (-)-Epigallocatechin-3-gallate decreases thrombin/paclitaxel-induced endothelial tissue factor expression via the inhibition of c-Jun terminal NH2 kinase phosphorylation. Biochem. Biophys. Res. Commun. 391, 716–721. 10.1016/j.bbrc.2009.11.126 19944065

[B41] Wang X. L. Fu A. Raghavakaimal S. Lee H. C. (2007). Proteomic analysis of vascular endothelial cells in response to laminar shear stress. Proteomics 7, 588–596. 10.1002/pmic.200600568 17309104

[B42] Wilson J. J. (2015). Antibody arrays in biomarker discovery. Adv. Clin. Chem. 69, 255–324. 10.1016/bs.acc.2015.01.002 25934364

[B43] Zhou Q. Desta T. Fenton M. Graves D. T. Amar S. (2005). Cytokine profiling of macrophages exposed to Porphyromonas gingivalis, its lipopolysaccharide, or its FimA protein. Infect. Immun. 73, 935–943. 10.1128/IAI.73.2.935-943.2005 15664935 PMC547047

