# Supplementary material for: Identification of Basic Fibroblast Growth Factor as the Dominant Protector of Laminar Shear Medium from the Modified Shear Device in Tumor Necrosis Factor-α Induced Endothelial Dysfunction
Source: Front Physiol. 2018 Jan 5;8:1095. doi: 10.3389/fphys.2017.01095 (PMC5760543; doi:10.3389/fphys.2017.01095)
Supplement: Supplementary file 1 [file DataSheet1.PDF]

Supplement data 2. Primers and Universal Probe Library probes list

GAPDH (#60):
Forward primer: 5’-CTCTGCTCCTCCTGTTCGAC-3’
Reverse primer: 5’-ACGACCAAATCCGTTGACTC-3’
VCAM-1 (#39):
Forward primer: 5’-TGCACAGTGACTTGTGGACAT-3’
Reverse primer: 5’-CCACTCATCTCGATTTCTGGA-3’
ICAM-1 (#71):
Forward primer: 5’-CCTTCCTCACCGTGTACTGG-3’
Reverse primer: 5’-AGCGTAGGGTAAGGTTCTTGC-3’
KLF-2 (#11):
Forward primer: 5’-CATCTGAAGGCGCATCTG-3’
Reverse primer: 5’-CGTGTGCTTTCGGTAGTGG-3’
HO-1 (#15):
Forward primer: 5’-GGCAGAGGGTGATAGAAGAGG-3’
Reverse primer: 5’-AGCTCCTGCAACTCCTCAAA-3’
NQO-1 (#21):
Forward primer: 5’-CAGCTCACCGAGAGCCTAGT-3’
Reverse primer: 5’-GAGTGAGCCAGTACGATCAGTG-3’
KEAP1 (#77):
Forward primer: 5’-GGGTCCCCTACAGCCAAG-3’
Reverse primer: 5’-TGGGGTTCCAGAAGATAAGC-3’
MCP-1 (#40):
Forward primer: 5’-GCCTCCAGCATGAAAGTCTC-3’
Reverse primer: 5’-GGAATGAAGGTGGCTGCTAT-3’
TM (#65):
Forward primer: 5’-AATTG GGAGC TTGGG AATG-3’
Reverse primer: 5’-TGAGG ACCTG ATTAA GGCTA GG-3’
TF (#5):
Forward primer: 5’-CCCCAGCTGTGTCAACTGT-3’
Reverse primer: 5’-CAGCACCATCCTTCAGACAC-3
PAI-1 (#19):
Forward primer: 5’-AAGGCACCTCTGAGAACTTCA-3’
Reverse primer: 5’-CCCAGGACTAGGCAGGTG-3’
EGFR (#21):
Forward primer: 5’-TTCCTCCCAGTGCCTGAA-3’
Reverse primer: 5’-GGGTTCAGAGGCTGATTGTG-3’
GM-CSF (#1):
Forward primer: 5’-TCTCAGAAATGTTTGACCTCCA-3’
Reverse primer: 5’-GCCCTTGAGCTTGGTGAG-3’
MIG (#4):

Forward primer: 5’-CCTTAAACAATTTGCCCCAAG-3’
Reverse primer: 5’-TTGAACTCCATTCTTCAGTGTAGC-3’
HGF (#15):
Forward primer: 5’-CAGCATGTCCTCCTGCATC-3’
Reverse primer: 5’-TCTTTTCCTTTGTCCCTCTGC-3’
GCSF (#1):
Forward primer: 5’-GAGCAAGTGAGGAAGATCCAG-3’
Reverse primer: 5’-CAGCTTGTAGGTGGCACACTC-3’
IL17A (#23):
Forward primer: 5’-CCCCAAAGCAGTTAGACTATGG-3’
Reverse primer: 5’-TTGAAGGATGAGGGTTCCTG-3’
bFGF (#4):
Forward primer: 5’-AGCGGCTGTACTGCAAAAAC-3’
Reverse primer: 5’-TGCTTGAAGTTGTAGCTTGATGT-3
