# Supplementary material for: Identification of Basic Fibroblast Growth Factor as the Dominant Protector of Laminar Shear Medium from the Modified Shear Device in Tumor Necrosis Factor-α Induced Endothelial Dysfunction
Source: Front Physiol. 2018 Jan 5;8:1095. doi: 10.3389/fphys.2017.01095 (PMC5760543; doi:10.3389/fphys.2017.01095)
Supplement: Supplementary file 2 [file DataSheet2.PDF]

Supplement data 3 The Array C6 and C7 databases of LSM and SM.

Array C6

Location of spots	Abbreviations	Cytokine name	Change fold (LSM/SM)	Up/ Down
F 1/2	ANG	Angiogenin	1.11	↑
G 1/2	BDNF	Brain-derived neurotrophic factor	0.75	↓
H 1/2	BLC	B lymphocyte chemoattractant	0.73	↓
I 1/2	BMP-4	Bone morphogenetic protein 4	0.88	↓
J 1/2	BMP-6	Bone morphogenetic protein 6	0.85	↓
K 1/2	CCL23	C-C motif chemokine 23	0.96	↓
L 1/2	CNTF	Ciliary neurotrophic factor	0.96	↓
M 1/2	EGF	Pro-epidermal growth factor	1.08	↑
N 1/2	Eotaxin-1	Eotaxin	0.76	↓
A 3/4	Eotaxin-2	C-C motif chemokine 24	1.15	↑
B 3/4	Eotaxin-3	C-C motif chemokine 26	0.91	↓
C 3/4	FGF-6	Fibroblast growth factor 6	0.85	↓
D 3/4	FGF-7	Fibroblast growth factor 7	0.84	↓
E 3/4	Flt-3 Ligand	Fms-related tyrosine kinase 3 ligand	0.83	↓
F 3/4	Fractalkine	Fractalkine	0.79	↓
G 3/4	GCP-2	Granulocyte chemotactic protein 2	0.71	↓
H 3/4	GDNF	Glial cell line-derived neurotrophic factor	0.86	↓
I 3/4	GM CSF	Granulocyte-macrophage colony-stimulating factor	0.45	↓
J 3/4	I-309	T lymphocyte-secreted protein I-309	0.65	↓
K 3/4	IFN gamma	Interferon gamma	0.73	↓
L 3/4	IGFBP- 1	Insulin-like growth factor-binding protein 1	0.85	↓
M 3/4	IGFBP- 2	Insulin-like growth factor-binding protein 2	0.97	↓
N 3/4	IGFBP- 4	Insulin-like growth factor-binding protein 4	1.07	↑
A 5/6	IGF-1	Insulin-like growth factor I	1.88	↑
B 5/6	IL-10	Interleukin-10	2.44	↑
C 5/6	IL-13	Interleukin-13	0.96	↓
D 5/6	IL-15	Interleukin-15	0.97	↓
E 5/6	IL-16	Interleukin-16	0.95	↓
F 5/6	IL-1 alpha	Interleukin-1 alpha	0.91	↓
G 5/6	IL-1 beta	Interleukin-1 beta	0.94	↓
H 5/6	IL-1 ra	Interleukin-1 receptor antagonist protein	1.02	↑
I 5/6	IL-2	Interleukin-2	0.72	↓
J 5/6	IL-3	Interleukin-3	0.74	↓
K 5/6	IL-4	Interleukin-4	0.82	↓
L 5/6	IL-5	Interleukin-5	0.78	↓

M 5/6	IL-6	Interleukin-6	0.88	↓
N 5/6	IL-7	Interleukin-7	0.86	↓
A 7/8	Leptin	Leptin	1.22	↑
B 7/8	LIGHT	Tumor necrosis factor ligand superfamily member 14	0.94	↓
**C 7/8**	**MCP-1**	**Monocyte chemoattractant protein 1**	**0.75**	↓
D 7/8	MCP-2	Monocyte chemoattractant protein 2	0.82	↓
E 7/8	MCP-3	Monocyte chemoattractant protein 3	0.68	↓
F 7/8	MCP-4	Monocyte chemoattractant protein 4	0.78	↓
G 7/8	M-CSF	Macrophage colony-stimulating factor 1	0.92	↓
H 7/8	MDC	Macrophage-derived chemokine	0.85	↓
I 7/8	MIG	Monokine induced by interferon-gamma	0.48	↓
J 7/8	MIP-1 delta	Macrophage inflammatory protein 1 delta	0.66	↓
K 7/8	MIP-3 alpha	Macrophage inflammatory protein 3 alpha	0.65	↓
L 7/8	NAP-2	Neutrophil-activating peptide 2	0.84	↓
M 7/8	NT-3	Neurotrophin-3	0.95	↓
N 7/8	PARC	Pulmonary and activation-regulated chemokine	0.93	↓
A 9/10	PDGF-BB	Platelet-derived growth factor subunit B	1.21	↑
B 9/10	RANTES	C-C motif chemokine 5	1.01	↑
C 9/10	SCF	Stem cell factor	0.81	↓
D 9/10	SDF-1alpha	Stromal cell-derived factor 1 alpha	0.90	↓
E 9/10	TARC	Thymus and activation-regulated chemokine	0.89	↓
F 9/10	TGF beta 1	Transforming growth factor beta-1	0.84	↓
G 9/10	TGF beta 3	Transforming growth factor beta-3	0.81	↓
H 9/10	TNF alpha	Tumor necrosis factor alpha	0.56	↓
I 9/10	TNF beta	Tumor necrosis factor beta	0.76	↓

C7 Cytokine antibody array

Location of spots	Abbreviations	Cytokine name	Change fold (LSM/SM)	Up/ Down
F 1/2	Acrp30	Adiponectin	1.39	↑
G 1/2	AgRP	Agouti-related protein	2.09	↑
H 1/2	ANGPT2	Angiopoietin-2	1.67	↑
I 1/2	AREG	Amphiregulin	3.08	↑
J 1/2	Axl	Tyrosine-protein kinase receptor UFO	4.83	↑
**K1/2**	**bFGF**	**Fibroblast growth factor 2**	**1.72**	↑
L 1/2	b-NGF	Beta-nerve growth factor	5.45	↑
M 1/2	BTC	Betacellulin	3.44	↑
N 1/2	CCL28	C-C motif chemokine 28	4.82	↑
A 3/4	CTACK	Cutaneous T-cell-attracting chemokine	1.05	↑
B 3/4	Dtk	Tyrosine-protein kinase receptor TYRO3	0.68	↓
C 3/4	EGFR	Epidermal growth factor receptor	0.48	↓

D 3/4	ENA-78	Epithelial-derived neutrophil-activating protein 78	0.87	↓
E 3/4	Fas	Tumor necrosis factor receptor family member 6	1.25	↑
F 3/4	FGF-4	Fibroblast growth factor 4	1.74	↑
G 3/4	FGF-9	Fibroblast growth factor 9	1.51	↑
H 3/4	G-CSF	Granulocyte colony-stimulating factor	9.61	↑
I 3/4	GITR Ligand	Glucocorticoid-induced TNF-related ligand	3.65	↑
J 3/4	GITR	Glucocorticoid-induced TNFR-related protein	4.17	↑
K 3/4	GRO	Growth-regulated protein	1.36	↑
L 3/4	GRO alpha	Growth-regulated alpha protein	1.36	↑
M 3/4	HCC-4	Chemokine CC-4	3.60	↑
N 3/4	HGF	Hepatocyte growth factor	23.70	↑
A 5/6	ICAM-1	Intercellular adhesion molecule 1	0.90	↓
B 5/6	ICAM-3	Intercellular adhesion molecule 3	0.77	↓
C 5/6	IGFBP 3	Insulin-like growth factor-binding protein 3	1.07	↑
D 5/6	IGFBP 6	Insulin-like growth factor-binding protein 6	1.21	↑
E 5/6	IGF-1R	Insulin-like growth factor 1 receptor	1.24	↑
F 5/6	IL-1R4	Interleukin-1 receptor-like 1	1.22	↑
G 5/6	IL-1R1	Interleukin-1 receptor type 1	1.21	↑
H 5/6	IL-11	Interleukin-11	2.65	↑
I 5/6	IL-12p40	Interleukin-12 subunit beta	2.30	↑
J 5/6	IL-12p70	Interleukin-12 subunit alpha	3.43	↑
K 5/6	IL-17	Interleukin-17A	6.39	↑
L 5/6	IL-2R alpha	Interleukin-2 receptor subunit alpha	6.32	↑
M 5/6	IL-6R	Interleukin-6 receptor subunit alpha	6.14	↑
N 5/6	IL-8	Interleukin-8	1.21	↑
A 7/8	I-TAC	Interferon-inducible T-cell alpha chemoattractant	1.10	↑
B 7/8	XCL1	XC chemokine ligand 1	1.24	↑
C 7/8	MIF	Macrophage migration inhibitory factor	1.64	↑
D 7/8	MIP-1 alpha	Macrophage inflammatory protein 1-alpha	1.15	↑
E 7/8	MIP-1 beta	Macrophage inflammatory protein 1-beta	1.18	↑
F 7/8	MIP-3 beta	Macrophage inflammatory protein 3-beta	1.21	↑
G 7/8	MSP alpha	Hepatocyte growth factor-like protein alpha chain	1.53	↑
H 7/8	NT-4	Neurotrophin-4	1.81	↑
I 7/8	OPG	Osteoprotegerin	2.05	↑
J 7/8	OSM	Oncostatin-M	1.40	↑
K 7/8	PLGF	Placenta growth factor	2.44	↑
L 7/8	gp130	Membrane glycoprotein 130	3.09	↑
M 7/8	TNF RII	Tumor necrosis factor receptor type II	2.96	↑
N 7/8	TNF RI	Tumor necrosis factor receptor type I	1.83	↑
A 9/10	TECK	Thymus-expressed chemokine	1.52	↑
B 9/10	TIMP-1	Tissue inhibitor of metalloproteinases 1	1.31	↑

C 9/10	TIMP-2	Tissue inhibitor of metalloproteinases 2	1.69	↑
D 9/10	THPO	Thrombopoietin	1.42	↑
E 9/10	TRAIL R3	TNF-related apoptosis-inducing ligand receptor 3	1.24	↑
F 9/10	TRAIL R4	TNF-related apoptosis-inducing ligand receptor 4	1.27	↑
G 9/10	uPAR	Urokinase plasminogen activator surface receptor	1.78	↑
H 9/10	VEGF	Vascular endothelial growth factor A	1.37	↑
I 9/10	VEGF-D	Vascular endothelial growth factor D	1.55	↑
