# Supplementary material for: Identification of Basic Fibroblast Growth Factor as the Dominant Protector of Laminar Shear Medium from the Modified Shear Device in Tumor Necrosis Factor-α Induced Endothelial Dysfunction
Source: Front Physiol. 2018 Jan 5;8:1095. doi: 10.3389/fphys.2017.01095 (PMC5760543; doi:10.3389/fphys.2017.01095)

Supplemental data 4

The serum levels of bFGF after TNF-α, TNF-α+LSM, TNF-α+rbFGF and TNF-α +LSM+Ab treatments were determined by ELISA (MFB00, R&D Systems). The highest bFGF level (381.12pg/mL) and the lowest level (219.36 pg/mL) were displayed in TNF-α+rbFGF and TNF-α +LSM+Ab groups, respectively. Data are expressed as mean ± S.E.M. (n=3). *$p < 0.05$ indicates a significant difference relative to the TNF-α group.

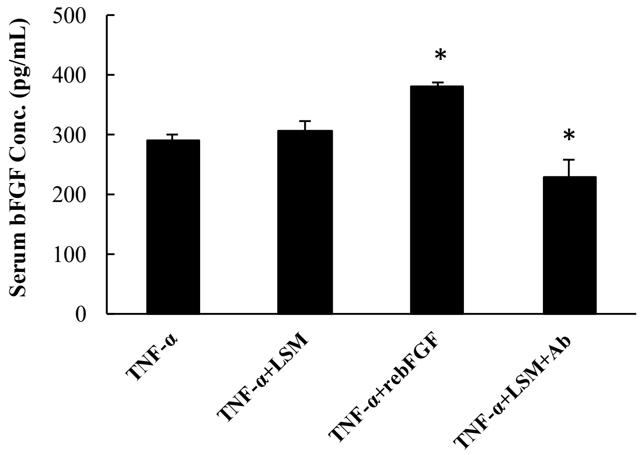

Supplement: Supplementary file 3 [file DataSheet3.PDF]
